# Airway host-microbiome interactions in chronic obstructive pulmonary disease

**DOI:** 10.1186/s12931-019-1085-z

**Published:** 2019-06-06

**Authors:** Zhang Wang, Barbara Maschera, Simon Lea, Umme Kolsum, David Michalovich, Stephanie Van Horn, Christopher Traini, James R. Brown, Edith M. Hessel, Dave Singh

**Affiliations:** 1 Computational Biology, Human Genetics, Research and Development (R&D), GlaxoSmithKline (GSK), 1250 S. Collegeville Road, Collegeville, PA 19426-0989 USA; 2 Present Address: School of Life Sciences, South China Normal University, Guangzhou, 510631 People’s Republic of China; 3 Refractory Respiratory Inflammation Discovery Performance Unit, Respiratory Therapy Area, R&D, GSK, Stevenage, SG1 2NY UK; 4 University of Manchester and University Hospital of South Manchester, Manchester, M23 9QZ UK; 5 Functional Genomics, Medicinal Science and Technology, R&D, GSK, Collegeville, PA 19426 USA

**Keywords:** Chronic obstructive pulmonary disease, COPD, Microbiome, Exacerbations, Clinical study, Transcriptome, Proteome, Healthy, Smokers, Next-generation sequencing technologies

## Abstract

**Background:**

Little is known about the interactions between the lung microbiome and host response in chronic obstructive pulmonary disease (COPD).

**Methods:**

We performed a longitudinal 16S ribosomal RNA gene-based microbiome survey on 101 sputum samples from 16 healthy subjects and 43 COPD patients, along with characterization of host sputum transcriptome and proteome in COPD patients.

**Results:**

Dysbiosis of sputum microbiome was observed with significantly increased relative abundance of *Moraxella* in COPD versus healthy subjects and during COPD exacerbations, and *Haemophilus* in COPD ex-smokers versus current smokers. Multivariate modeling on sputum microbiome, host transcriptome and proteome profiles revealed that significant associations between *Moraxella* and *Haemophilus*, host interferon and pro-inflammatory signaling pathways and neutrophilic inflammation predominated among airway host-microbiome interactions in COPD. While neutrophilia was positively correlated with *Haemophilus*, interferon signaling was more strongly linked to *Moraxella.* Moreover, while *Haemophilus* was significantly associated with host factors both in stable state and during exacerbations, *Moraxella*-associated host responses were primarily related to exacerbations.

**Conclusions:**

Our study highlights a significant airway host-microbiome interplay associated with COPD inflammation and exacerbations. These findings indicate that *Haemophilus* and *Moraxella* influence different components of host immune response in COPD, and that novel therapeutic strategies should consider targeting these bacteria and their associated host pathways in COPD.

**Electronic supplementary material:**

The online version of this article (10.1186/s12931-019-1085-z) contains supplementary material, which is available to authorized users.

## Background

Chronic obstructive pulmonary disease (COPD) is a heterogeneous lung disease in which recurrent bacterial infections are a major etiological factor [1–4]. The human microbiome in the respiratory tract differs between healthy subjects and COPD patients [5–7], shifts in composition during COPD exacerbations [8–12] and varies among exacerbation subtypes [9], all suggesting a close association between the lung microbiome and COPD pathophysiology with potential involvement of host immunity and inflammatory responses. It is thought that disruption of microbiome, known as dysbiosis, could trigger a dysregulated host immune response that results in infection susceptibility, inflammation and negative effects on host biology [13].

A systematic understanding of airway host-microbiome interaction in relation to COPD pathogenesis could provide the mechanistic basis for modulation of host-microbe interactions as a potential novel therapeutic strategy for COPD. A previous study on COPD patients showed that the lung microbiome was significantly associated with sputum pro-inflammatory markers especially interleukin-8 (IL8/CXCL-8, 9). In particular, there is a significant correlation between sputum interleukin-8 (IL-8/CXCL-8) with both alpha and beta diversity of the airway microbiome in COPD. In the correlation network, sputum IL-8/CXCL-8 showed the highest degree of microbiota connectivity with a significant negative correlation to 15 bacterial operational taxonomic units (OTUs), suggesting sputum IL-8/CXCL-8 could be an indicator of microbiome community structure and diversity.

Few studies have simultaneously characterized both lung microbiome and human multi-omics profiles in COPD, and in other respiratory diseases in general. Sze et al. measured the lung microbiome and host transcriptome in COPD and found Firmicutes and Proteobacteria were associated with different host gene expression profiles [14]. Molyneaux et al. profiled both lung microbiome and peripheral whole-blood transcriptome for idiopathic pulmonary fibrosis patients and identified two gene modules involved in host defense that are strongly associated with the microbiome profile [15] However, a comprehensive understanding of the collective host response at both transcriptional and protein expression levels to the lung microbiome community is lacking. A systems biology approach integrating lung microbiome and host multi-omics datasets is necessary to better understand host-microbiome interactions in COPD.

Here we performed a 16S ribosomal RNA (rRNA) gene-based survey on sputum microbiome from 16 healthy subjects and 43 COPD patients. Host sputum cell counts, transcriptome and proteome were also characterized for COPD patients. To our knowledge, this is the first study that characterizes both lung microbiome and host transcriptome and proteome profiles in stable COPD and during exacerbations. We found significant interplay between lung microbiome composition and host response in COPD that is potentially important to current treatments and future therapeutic strategies.

## Methods

### Patient selection

The presented study was conducted in accordance with the Declaration of Helsinki [16] and Good Clinical Practice [17]. The human biological samples were sourced ethically and in accord with the terms of the informed consents under the University of Manchester and University Hospital of South Manchester IRB/EC approved protocol (Approval number: 10/H1003/108).

Healthy subjects and COPD patients were enrolled at the Medicines Evaluation Unit (Manchester University Foundation NHS Trust Hospital). Patients with asthma, or significant respiratory disease other than COPD, or the inability to produce sputum after sputum induction were excluded from the study. Patients were seen at stable at least 6 weeks after the use of any short term antibiotics. Patients contacted the research team if they experienced a change in symptoms consistent with an acute exacerbation. Daily diary cards were used. Patients were assessed by a clinician and exacerbations defined as in increase in respiratory symptoms for two consecutive days. Smoking status, historical exacerbation frequency, GOLD status, inhaled corticosteroid (ICS) administration, Quality of Life (QoL) scores and lung function measurements (FEV_1_, FVC and FEV_1_/FVC ratio) were recorded for COPD patients (Table 1, Additional file 1: Table S1). Smoking status and lung function measurements were recorded for healthy subjects.

**Table 1:** Major demographic and baseline clinical features of all subjects in this study

Demographic and baseline clinical features	Healthy Controls (*N* = 16)	COPD Patients (*N* = 43)
Age, years^a^	55.0 (8.8)	65.0 (4.8)
Gender, *n* (% Male) ^b^	11 (68.8)	31 (72.1)
Current Smoker, *n* (%)	8 (50.0)	16 (37.2)
Number of cigarette packs per year	34.4 (11.2)	50.9 (28.2)
GOLD Stage: I/II/III/IV, *n* (%)	NA	2 (4.7)/19 (44.2)/16 (37.2)/6 (14.0)
Inhaled steroid use, *n* (%)	NA	33 (76.7)
LABA use, *n* (%)	NA	34 (79.1)
LAMA use, *n* (%)	NA	35 (81.4)
Number of exacerbations per year	NA	1.9 (1.4)
CAT score	NA	21.8 (8.3)
SGRQ total score	NA	49.0 (22.9)
mMRC score	NA	2.1 (1.2)
Pre FEV_1_ (L)	3.0 (0.7)	1.1 (0.5)
Pre FVC (L)	4.1 (1.0)	2.8 (0.9)
Pre FEV_1_/FVC ratio	0.73 (0.0)	0.4 (0.1)
Post FEV_1_ (L)	3.1 (0.8)	1.2 (0.6)
Post FVC (L)	4.0 (0.9)	3.3 (1.0)
Post FEV_1_/FVC ratio	0.76 (0.0)	0.4 (0.1)

^a^ Continuous data present as mean (SD)

^b^ Categorical data present as number (proportion)

*GOLD* Global Initiative for Chronic Obstructive Lung Disease, *LABA* long-acting beta-agonist, *LAM* long-acting muscarinic antagonist, *CAT* COPD Assessment Test, *SGRQ* St. George’s Respiratory Questionnaire, *mMRC* modified Medical Research Council, *FEV*_*1*_ forced expiratory volume in one second, *FVC* forced vital capacity

### Sputum collection

Sputum samples were collected at a single time-point from 16 healthy subjects and longitudinally from 43 COPD patients. Sputum sampling were performed prior to any systemic therapy including treatment with oral corticosteroids and/or antibiotics. Sputum samples were obtained by spontaneous expectoration or induced. For COPD patients, spontaneous expectoration was attempted first, if no sputum or too little sputum was produced, induction was then performed. For healthy subjects, only induction method was performed. Sputum samples from COPD patients were collected at stable (defined as no evidence of symptom-defined exacerbations in the preceding 4 weeks and the subsequent 2 weeks post-clinic visit), exacerbations (defined according to Anthonisen criteria [18] and/or healthcare utilization [19]), two and 6 week post-exacerbations and 6 months from first stable visit. All exacerbation sputum samples were collected prior to the institution of any exacerbation treatment. The missing samples are mostly due to patients unable to produce sufficient amount of sputum for downstream experiments (Additional file 1: Figure S1).

### Sputum processing

Sputum samples were processed to obtain cell pellets and supernatant, for immune cell counting, host transcriptome and proteome analysis, according to a previous method [20]. Briefly, sputum plugs were selected from saliva and put on ice (minimum weight 0.1 g). Eight times volume of phosphate-buffered saline (PBS) was added to the sputum. The mixture was incubated in a roller mixer for 15 min on ice, vortexed every 5 min and centrifuged at 790 g for 10 min. The supernatant was split into aliquots and stored at − 80 °C for sputum proteome analysis. For cell pellets, a four-fold volume of 0.2% DTT was added and the mixture was incubated for 15 min in a roller mixer on ice, vortexed every 5 min, filtered using 48 μm nylon-mesh filter and centrifuged. Cell pellets were resuspended in 1 ml of PBS to perform haemocytometer cell counts, cytospin differential cell counts and stored at − 80 °C for transcriptomic assays.

### Microbiome 16S rRNA gene sequencing

For quality control purposes, bacterial DNA extractions, sequencing and data analyses were performed in a single, centralised lab at the GlaxoSmithKline (GSK) R&D facility in Collegeville, PA, USA. The detailed procedure of bacterial genomic DNA isolation, 16S library preparation, sequencing, reagent controls, and sequence data processing was provided in the supplementary material of our previous study [11]. Bacterial genomic DNA was extracted from healthy and COPD sputum samples using Qiagen DNA Mini kit. The variable 4 (V4) region of the 16S rRNA gene was PCR-amplified with the appropriate reagent controls [9, 11], and was sequenced using Illumina Miseq. The demultiplexed and quality-filtered sequencing reads were subject to open-reference operational taxonomic unit picking (97% identity cutoff) using QIIME 1.9 [21].

Seven OTUs were detected with > 10 sequencing reads in the negative reagent controls (Additional file 1: Table S2). Although negative reagent controls were performed for all DNA isolation, extraction and PCR amplification step, we performed further analyses to ensure that potential contamination risks were minimized. We compared our results against the 92 contaminant genera detected in sequenced negative ‘blank’ controls by Salter et al. [22]. We failed to detect 56 out of the 92 contaminant genera in our dataset (Additional file 1: Table S3). Of the remaining genera that were found in our data, none had an average relative abundance greater than 0.0004, or had a relative abundance greater than 0.1 in a particular sample, except for *Streptococcus* which contains known lung pathogens.

### Bacterial qPCR assays

All qPCR assays were performed using 384-well microbial DNA qPCR arrays (Qiagen, Germantown, MD) on a QuantStudio 12 K Flex Real-Time PCR System (Life Technologies, Carlsbad, California, USA). The 10 μl reaction mixture contained 5 μl of Microbial qPCR master mix with ROX and 5 μl of Microbial-free water (Qiagen, Germantown, MD). Each well was spotted with a mix of two PCR primers (10 μM each) and one 5′-hydrolysis probe (5 μM) with 10 ng of added sample DNA. The following cycling parameters were used: initial cycle of 95 °C for 10 min; 40 cycles of 95 °C for 15 s; and 60 °C for 2 min. All qPCR templates were run in duplicate and tested for amplification inhibition by use of a positive PCR Control (Qiagen, Germantown, Maryland, USA). For standard curve calculation, each plate run included a decimal serial dilution of double-stranded DNA oligos (Integrated DNA Technologies, Skokie, Illinois, USA) designed from the 16S rRNA gene of pan-bacteria, *Haemophilus influenzae*, *Moraxella catarrhalis*, *Streptococcus pneumoniae*, *Prevotella melaninogenica* and *Veillonella dispar*. The cycle threshold values and DNA copy numbers were calculated using the QuantStudio 12 K Flex software (Life Technologies, Carlsbad, California, USA).

### Host RNA microarray analysis

Host transcriptome was profiled for 38 COPD sputum samples (Additional file 1: Figure S1). Total RNA was extracted using Trizol reagent (Invitrogen) from sputum cell pellets and further purified with a RNeasy mini kit (Qiagen, Valencia, California, USA) according to the manufacturer’s instructions. RNA quality was evaluated on the Agilent 2100 Bioanalyzer and quantitated by OD260. For samples passing RNA QC criteria (RIN > 5.5, A260/280 value 1.6–2.4, total RNA > 50 ng, presence of distinct 28S and 18S ribosomal RNA peaks), 50 ng RNA was used for NuGEN amplification and labeling of probes using the NuGEN Ovation RNA Amplification System (NuGEN Technologies). The amplified sscDNA was purified using the Agencourt RNAClean magnetic bead clean-up system. The sscDNA samples were quantified by spectrophotometry and profiled on Agilent 2100 Bioanalyser prior to array hybridization. The array hybridization was performed using Affymetrix GeneChip HG-U133 Plus 2.0 microarray (Affymetrix, Santa Clara, California, USA), which contains 54,675 probe-sets interrogating 50,155 human transcripts. The raw microarray data (CEL files) were corrected for background signal, quantile normalized and summarized using robust multiarray average (RMA) normalization to generate probe-set-level microarray data using Array Studio v10.0 (OmicSoft, Cary, North Carolina, USA). The probe-set-level microarray data were log2 transformed and converted to gene-level (24,442 genes) by selecting the probe with greatest inter-quantile range for its corresponding gene as suggested previously [23]. The microbiome and microarray data are deposited at the National Centre for Biotechnology Information Sequence Read Archive (SRP136124) and Gene Expression Omnibus databases (GSE112165), respectively.

### Proteomic assays

Host proteome was characterized for 37 sputum samples using the SOMAscan® platform (Somalogic, Additional file 1: Figure S1). The SOMAscan® assay has been described in detail previously [24–26]. The assay quantitatively transforms the proteins present in a biological sample into a specific SOMAmer-based DNA signal. Briefly, each SOMAmer® reagent binds a target protein (in total 1310 proteins) and is quantified on a custom Agilent microarray hybridization chip. Normalization and calibration were performed according to SOMAscan® Data Standardization and File Specification Technical Note (SSM-020). The output of the SOMAscan® assay was reported in relative fluorescent units and was log2 transformed for downstream analysis.

### Statistical analysis

Differentially represented bacterial taxa were identified using edgeR [27]. Differentially expressed genes in microarray were identified using limma in R bioconductor [28] and were enriched for pathways using MetaCore v5.0 (Thomson Reuters). Multivariate modeling was performed to associate microbiome, host transcriptome and proteome data. The power calculation was performed following the procedure of Morgan et al. [29]. Specifically, correlated variable pairs were simulated with standard normal distribution and a sample size of 40, the number of samples that have both transcriptome/proteome and microbiome data. The 80th percentile of raw *P*-values of the Spearman correlation test was calculated as a function of true covariance of the variables. The number of allowable tests for 80% power and 5% type I error rate was estimated by Bonferroni correction, which is 0.05 divided by the 80th percentile of raw *P*-values calculated as above.

To reduce dimensionality, a Principal Component Analysis (PCA) was performed on the gene-level microarray data. PCA was also performed on the Somalogic proteome profile of 1310 proteins. The transcriptome Principal Components (tPCs) and proteomic Principal Components (pPCs) with proportion of variance > 2% were selected for association testing. For the microbiome datasets, 9 bacterial genera with average relative abundance > 1% were selected for association testing. A variance-stabilizing arcsin square root transformation was applied to the microbiome proportion data. All continuous variables were scaled to unit variance. For each genus and Shannon diversity, a general linear mixed model (GLMM) was established associating the variable with tPCs and pPCs adjusting for timepoints and patient demographic factors including smoking status, GOLD status and exacerbation frequency using lme4 in R [30]. Subject ID was included as a random variable to adjust for multiple measures per subject. The model was optimized in terms of Akaike information criterion (AIC) through backward elimination of non-significant effects in a stepwise algorithm using the “step” function in the R lmerTest package [31]. The same GLMM was applied for associating each pPCs with all tPCs. For association within stable samples, as no repeated measures were involved, a general linear model (GLM) was established using glm in R [32]. The model was optimized in terms of AIC through backward elimination of non-significant effects in a stepwise algorithm using the “step” function in the R stats package [32].

To assess functional enrichment of each tPC, all 24,442 genes were ranked by their loadings in that tPC and a Gene Set Enrichment Analysis (GSEA) [33] was performed on the ranked gene list using concatenated MetaCore (GeneGo), KEGG, Reactome, BioCarta and Pathway Interaction Database (PID) pathways (a total of 2809 pathways) using GSEAP reranked 6.0.10 in GenePattern [34]. The enrichment scoring scheme was set to ‘classic’ as suggested in the program instructions. One thousand permutations were performed for each run. Gene sets larger than 500 genes or smaller than 15 genes were excluded from the analysis.

The false discovery rate (FDR) method was used to adjust *P*-values for multiple testing wherever applicable [35].

## Results

### Sputum microbiome between healthy and COPD and during exacerbations

**Fig. 1:**
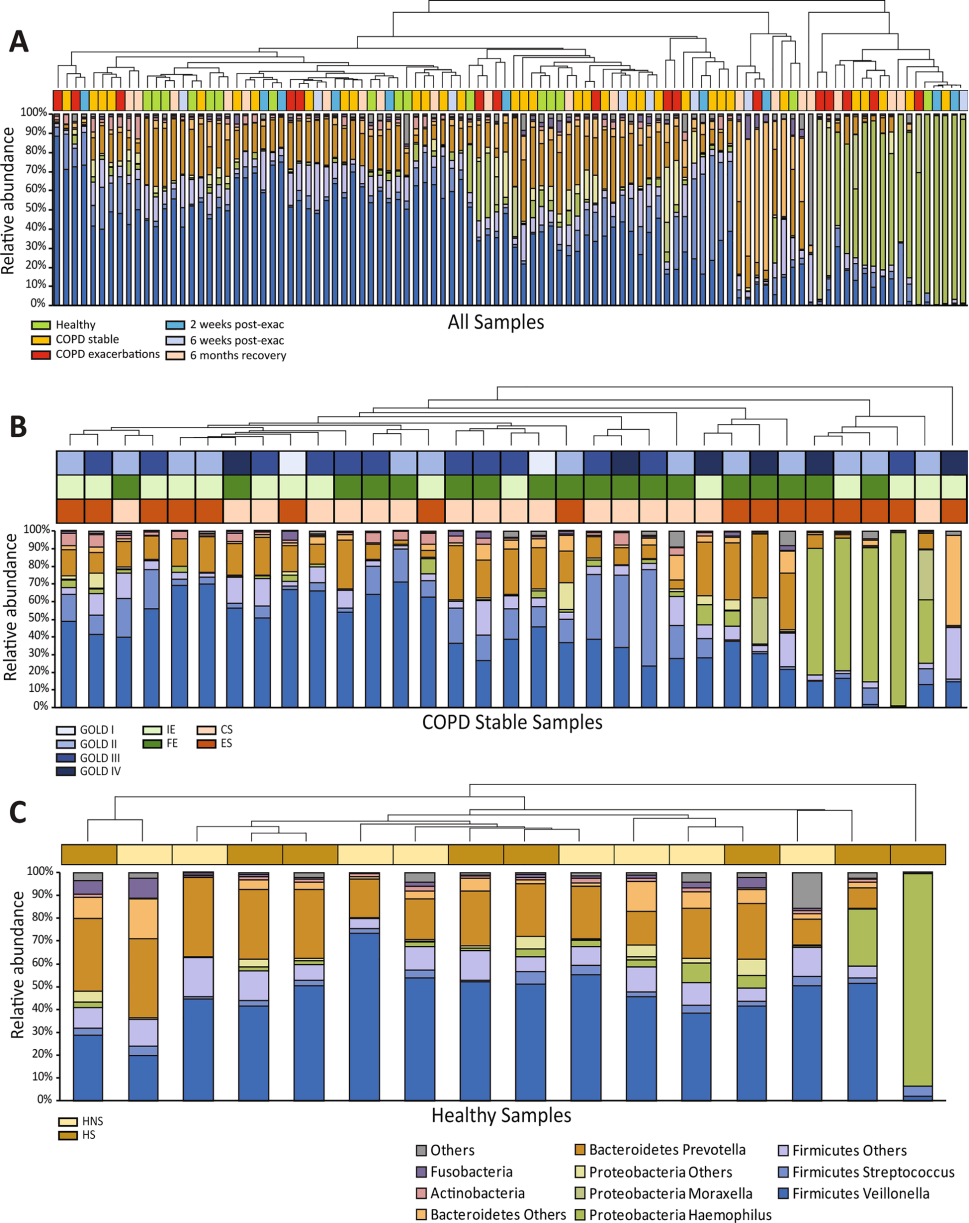
Overview of the sputum microbiome taxa distributions. **a** Overall study clustering for all 101 samples. **b** Clustering of 32 COPD stable samples. **c** Clustering of 16 healthy samples. Each column represents one sample colored by different subgroups. Y-axis represents relative abundances of major phyla and genera. Samples were clustered by UPGMA clustering based on the weighted UniFrac distances. HNS: healthy non-smokers, HS: healthy smokers, CS: COPD current smokers, ES: COPD ex-smokers, non-ICS: non-ICS exposer, ICS: ICS exposer, IE: infrequent exacerbators, FE: frequent exacerbators

Sputum microbiome was characterized for 101 sputum samples (Fig. 1a) from COPD patients (Fig. 1b) and healthy controls (Fig. 1c). A total of 16,386,538 reads were generated after demultiplexing and quality filtering. After rarefaction to 89,462 reads per sample, 2807 OTUs were identified among all samples. Similar to other lung microbiome studies [5, 6, 8–10, 36–39], the majority of OTUs belonged to Firmicutes (53.6%), Bacteroidetes (21.9%) and Proteobacteria (19.5%) at the phylum level, and *Veillonella* (37.7%), *Prevotella* (15.3%), *Haemophilus* (14.0%), *Streptococcus* (8.6%) and *Moraxella* (2.9%) at the genus level. Quantitative PCR showed significant correlations between the absolute quantities of all five species and their relative abundances in the microbiome data (Spearman’s rho ≥0.43, FDR *P* = 2.57E-3).

**Fig. 2:**
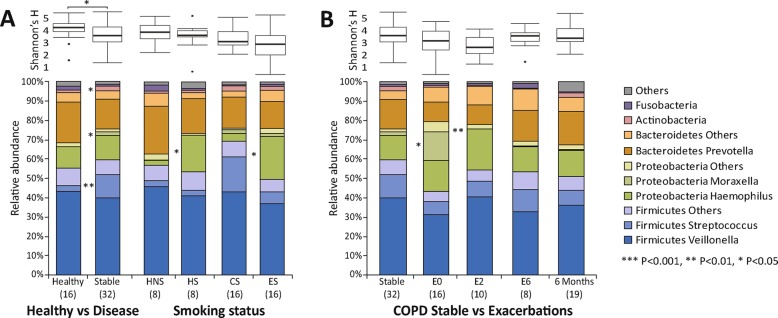
Sputum microbiome profiles in healthy subjects and COPD patients. **a** Shannon diversity and relative abundance of major bacterial taxa in healthy controls and stable COPD patients, and in healthy and COPD subgroups in relation to smoking status. **b** Shannon diversity and relative abundances of major bacterial taxa in COPD patients at different visits. The number of samples in each group is indicated in the parenthesis. Significantly differentially represented bacterial taxa were identified using edgeR [27]. For visit, statistical analysis was performed on each adjacent two time points. E0: COPD exacerbations, E2: 2 week post-exacerbations, E6: 6 week post-exacerbations, 6 Months: 6 months from first stable visit, HNS: healthy non-smokers, HS: healthy smokers, CS: COPD current smokers, ES: COPD ex-smokers. *** FDR *P* < 0.001, ** FDR *P* < 0.01, * FDR *P* < 0.05

Significantly increased relative abundance of *Haemophilus* was observed in healthy smokers versus non-smokers (log_2_FC = 3.36, FDR *P* = 0.041), and in COPD ex-smokers versus current smokers (log_2_FC = 2.49, FDR *P* = 0.025, Fig. 2a). Comparison of the microbiome profiles between healthy subjects and stable COPD patients showed a significantly increased relative abundance of the genera *Moraxella*, *Streptococcus* and Actinobacteria (log_2_Fold Change (log_2_FC) ≥ 1.32, FDR *P* = 0.026, Additional file 1: Table S4) and decreased alpha diversity (Shannon, *P* = 0.036) in stable COPD patients (Fig. 2b). A significantly increased *Moraxella* was observed at stable state in GOLD III versus II patients and in inhaled corticosteroids (ICS) versus non-ICS exposed patients (Additional file 1: Figure S2).

During COPD exacerbations, increased *Moraxella* (log_2_FC = 3.14, FDR *P* = 0.019) and decreased alpha diversity was observed compared to stable state (unpaired analysis, Fig. 2b, paired analysis see Additional file 1: Figure S3), along with significantly increased neutrophil and decreased macrophage percentage (FC ≥ 1.2, *P* ≤ 0.05, Additional file 1: Figures S4–S5). A non-significant increase of total bacterial load was observed during exacerbations (Additional file 1: Figure S6). Conversely, the trend of increased *Moraxella* and decreased alpha diversity was reversed at post-exacerbation time points (Fig. 2b).

**Fig. 3:**
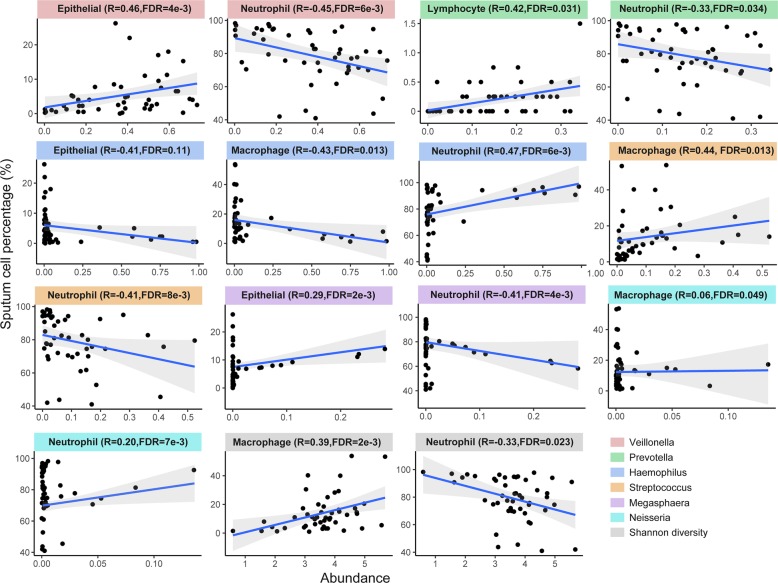
Significant spearman correlations (with 95% confidence intervals calculated by univariate regression model) between major sputum microbiome compositions with sputum leukocyte percentages

Sputum neutrophil counts were most significantly associated with microbiome compositions, with positive correlations with *Haemophilus* and *Neisseria*, and negative correlations with *Streptococcus*, *Megasphaera* and *Veillonella* across all samples (Spearman’s rho = 0.33, FDR *P* ≤ 0.05, Fig. 3, Additional file 1: Table S5). The significant correlation between *Haemophilus* and sputum neutrophil count was further confirmed by qPCR (Spearman’s rho = 0.37, *P* = 0.037, Additional file 1: Table S6). No bacterial taxa or sputum cell counts were associated with QoL scores, FEV_1_ or FVC.

### Host transcriptome and proteome at COPD stable state and exacerbations

We compared host transcriptome differences between COPD stable and exacerbations. A substantial amount of 2453 upregulated and 4814 downregulated differentially expressed genes (DEGs) were identified at exacerbations versus stable state (FC ≥ 1.5, FDR *P* ≤ 0.05), in which 239 and 8 MetaCore pathways were significantly enriched respectively (FDR *P* ≤ 0.01, Additional file 2). A large proportion of the upregulated pathways were involved in immune response with the top pathways being interferon and interleukin-6 signaling pathways. The downregulated pathways included cell cycle, nucleotide metabolism and phagocytosis pathways. No DEGs were found between stable patient subgroups according to clinical characteristics (GOLD stage, smoking status, ICS administration and exacerbation frequency).

For patient proteome data, 790 of the 1310 proteins had significantly higher expression levels in stable COPD ex-smokers compared to current smokers, including multiple pro-inflammatory markers such as interleukin-36, fibrinogen and matrix metallopeptidase 10 (FC ≥ 1.5, FDR *P* ≤ 0.05, Mann-Whitney-Wilcoxon test, Additional file 2). No differentially expressed proteins were identified for other comparisons.

### *Haemophilus* and *Moraxella* are most significantly associated with host transcriptome and proteome

**Fig. 4:**
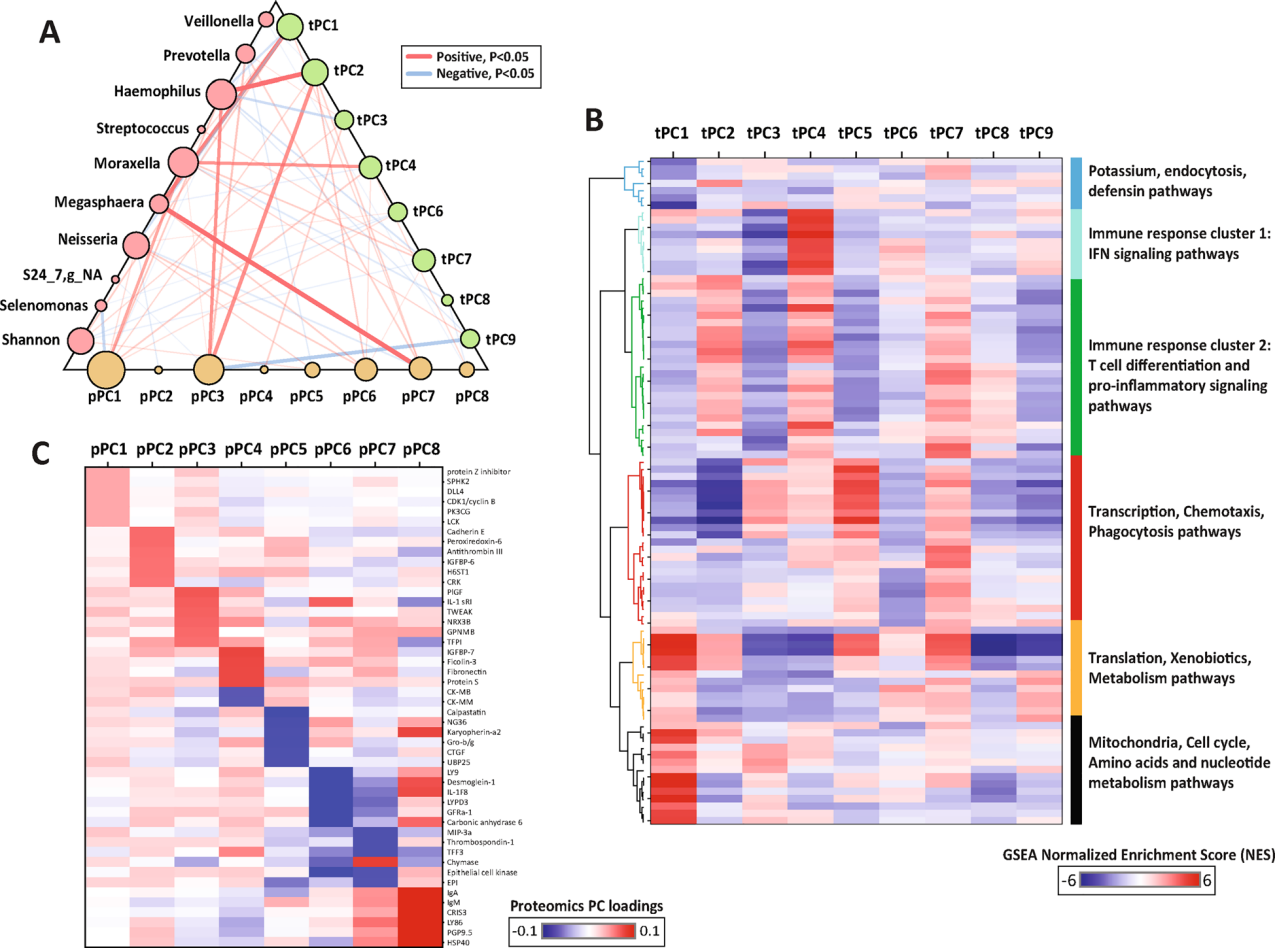
Multivariate modeling showed strong association of *Haemophilus* and *Moraxella* with host transcriptome and proteome profiles. **a** A host-microbiome interaction network illustrating significant associations among the 9 most abundant bacterial genera and Shannon diversity, tPCs and pPCs in GLMM. Each edge indicates a significant association (FDR *P* ≤ 0.05) colored by direction. The edge weight corresponds to the significance of the *P*-value. The size of the node is proportional to the number of significant associations involving the node. **b** GSEA enrichment scores of the top pathways on the loadings of each tPC. For each tPC, the top 10 positively and negatively enriched pathways (FDR *P* ≤ 0.01) were included in the heatmap. Pathways were clustered using complete clustering and colored by their clustering groups. The functional categories of the pathways are overall in agreement with their clustering groups. **c** Top loadings of each pPC. For each pPC, the top 6 proteins by magnitude of loadings were included in the heatmap

To gain insights into airway host-microbiome interactions in COPD, we established a multivariate linear model between microbiome, host transcriptome and proteome profiles across all samples (including exacerbations) and within stable samples only. We first performed a power estimation and calculated that given a true covariance of 0.5 between bacterial taxa and gene expression in 40 samples (the number of samples with both transcriptome/proteome and microbiome data), it would be possible to perform a maximum of 10^2^ pairwise tests (or approximately 10 microbiome and 10 host expression factors) and retain 80% power and an alpha of 0.05 using Bonferroni correction (Additional file 1: Figure S7). As it is impossible for significant associations to survive correction for multiple testing of ~ 20,000 human genes, we performed an unsupervised dimensionality reduction on host multi-omics data using PCA. A total of 9 transcriptome and 8 proteome PCs (tPCs and pPCs, respectively) with proportion of variance > 2% were selected, together explaining 72 and 84% of observed variance. Using all samples (including exacerbations), a GLMM was established between each of the 9 major bacterial genera and all tPCs or pPCs, adjusting for different timepoints and patient demographic factors. Among all genera, *Haemophilus* and *Moraxella* were most strongly associated with host factors, in particular strong positive correlations with tPC2 and tPC4, respectively (FDR *P* < 5.0E-4, Fig. 4a, Table 2, Additional file 3).

**Table 2:** Associations of the 9 bacterial genera and Shannon diversity with tPCs both across all samples and within stable samples in generalized linear mixed models. FDR *P*-values are indicated in the table. Significant associations are highlighted in asterisks. Only significant variables were included in the final model unless otherwise stated

	Microbiome	Transcriptome								
		tPC1	tPC2	tPC3	tPC4	tPC5	tPC6	tPC7	tPC8	tPC9
All	*Veillonella*	0.57	0.05*	0.91	0.39	0.32	0.53	0.71	0.82	0.70
	*Prevotella*	0.34	0.42	0.04*	0.25	0.93	0.92	0.59	0.59	0.14
	*Haemophilus*	0.01*	1E-4*	3E-3*	0.97	0.68	0.41	0.01*	0.33	0.02*
	*Streptococcus*	0.19	0.17	0.29	0.67	0.59	0.87	0.03*	0.36	0.36
	*Moraxella*	0.03*	0.45	0.88	5E-4*	0.09	0.30	0.01*	0.02*	0.02*
	*Megasphaera*	2E-4*	0.95	0.15	0.03*	0.28	0.78	0.91	0.84	0.79
	*Neisseria*	5E-3*	0.92	0.26	0.29	0.21	0.05*	0.19	0.18	0.23
	*S24–7*	0.72	0.73	0.38	0.52	0.94	0.84	0.80	0.57	0.46
	*Selenomonas*	0.29	0.97	0.13	0.24	0.09	0.04*	0.33	0.21	0.48
	Shannon	0.01*	0.10	0.02*	0.91	0.51	0.03*	0.04*	0.06	0.92
Stable	*Veillonella*	0.27	0.06	0.05	0.25	0.26	0.95	0.08	0.83	0.53
	*Prevotella*	0.37	0.36	0.27	0.51	0.56	0.67	0.27	0.90	0.27
	*Haemophilus*	0.10	0.01*	0.04*	0.36	0.76	0.62	0.16	0.31	0.51
	*Streptococcus*	0.80	0.64	0.73	0.28	0.56	3E-3*	0.08^a^	0.86	0.51
	*Moraxella*	0.40	0.59	0.81	0.46	0.57	0.35	0.75	0.13	0.24
	*Megasphaera*	0.58	0.43	0.02*	0.50	0.39	0.07^a^	0.45	0.67	0.78
	*Neisseria*	0.73	0.49	0.95	0.44	0.09^a^	0.60	0.50	0.83	0.60
	*S24–7*	0.39	0.73	0.52	0.82	0.42	0.90	0.03*	0.72	2E-3*
	*Selenomonas*	0.02*	0.59	0.50	0.58	0.90	0.92	0.57	0.88	0.56
	Shannon	0.06	0.10	0.08	0.33	0.26	0.37	0.92	0.07	0.26

^a^Variables not statistically significant but present in the model

Examining the top loadings of tPC2 and tPC4 revealed that they reflected increased expression of some immune response genes, such as interleukin-1 receptor-associated kinase 1 (*IRAK1*), interleukin-18 binding protein (*IL18BP*), linker for activation of T-cells family member 1 (*LAT*) for tPC2, and several interferon genes for tPC4 (Additional file 1: Figure S8, Additional file 4), indicating that high level of these two tPCs might correspond to increased immune activities. We performed GSEA on the loadings of each tPC to further understand its functional properties. Both tPC2 and tPC4 were most significantly positively enriched for host immune response pathways. Several T-cell differentiation (i.e. Th1 and Th2 cell differentiation) and pro-inflammatory cytokine (i.e. IL-12) signaling pathways were among the top positive pathways for tPC2, while interferon signaling pathways were the top positive pathways for tPC4 (Fig. 4b, Additional file 4). Individual genes in the top pathways of tPC2 and tPC4 showed consistent correlations with *Haemophilus* and *Moraxella* respectively in both microbiome and qPCR datasets (Additional file 5), further supporting the associations in the multivariate models. Furthermore, both *Haemophilus* and tPC2 exhibited positive correlations with pPC3 (FDR *P* = 3.1E-3, Table 3, Additional file 3), together forming an interconnected subnetwork (Fig. 4a). Likewise, both *Moraxella* and tPC4 were positively correlated with pPC1 (FDR *P* = 0.014, Additional file 3). Multiple pro-inflammatory markers such as interleukin-1 receptor, matrix metalloproteinase 7, galectin-2 and TNF-related weak inducer of apoptosis were among the top loadings for pPC1 or pPC3 (Fig. 4c). In addition, several known bronchial epithelial cell receptors that respond to bacterial lipopolysaccharide (LPS) such as angiopoietin-1 receptor [40] and Ephrin-A2 [41] were among the top loadings for the two pPCs (Additional file 4). The correlations of *Haemophilus*-tPC2-pPC3 and *Moraxella-*tPC4-pPC1 were further confirmed by qPCR (*P* ≤ 0.1, Additional file 1: Table S6). In addition, both tPC4 and pPC1 were increased at exacerbations versus stable (Additional file 1: Figure S9). Both tPC2 and pPC3 were significantly positively correlated with sputum neutrophil counts (FDR *P* ≤ 0.05, Additional file 1: Table S5).

**Table 3:** Associations of the 9 bacterial genera and Shannon diversity with pPCs both across all samples and within stable samples in generalized linear mixed models. FDR *P*-values are indicated in the table. Significant associations are highlighted in asterisks. Only significant variables were included in the final model unless otherwise stated

	Microbiome	Proteome							
		pPC1	pPC2	pPC3	pPC4	pPC5	pPC6	pPC7	pPC8
All	*Veillonella*	0.04*	0.82	0.54	0.62	0.40	0.19	0.62	0.04*
	*Prevotella*	0.52	0.94	0.01*	0.11	0.02*	0.76	0.01*	0.34
	*Haemophilus*	0.01*	0.06	1E-3*	0.69	0.25	0.47	0.64	0.56
	*Streptococcus*	0.29	0.35	0.92	0.69	0.45	0.93	0.13	0.61
	*Moraxella*	1E-3*	0.54	0.05^a^	0.45	0.13	0.40	0.85	0.52
	*Megasphaera*	0.31	0.36	0.06^a^	0.67	0.51	0.01*	1E-4*	0.84
	*Neisseria*	0.03*	0.06^a^	0.22	0.26	0.02*	0.01*	0.02*	0.06
	*S24–7*	0.67	0.12	0.04*	0.24	0.21	0.58	0.49	0.55
	*Selenomonas*	2E-3*	0.14	0.58	0.12	0.72	0.69	0.13	0.29
	Shannon	0.04*	0.54	0.78	0.10	0.03*	0.56	0.58	0.09
Stable	*Veillonella*	0.05^a^	0.10	0.10	0.23	0.71	0.47	0.99	0.34
	*Prevotella*	0.73	0.05^a^	0.21	0.03*	0.86	0.38	0.70	0.50
	*Haemophilus*	0.03*	0.03*	2E-3*	0.08	0.75	0.29	0.73	0.60
	*Streptococcus*	0.03*	0.93	0.58	0.35	0.76	0.82	0.62	0.24
	*Moraxella*	0.46	0.97	0.26	0.59	0.95	0.96	0.30	0.79
	*Megasphaera*	0.38	0.76	0.80	0.69	0.80	0.27	0.67	0.25
	*Neisseria*	0.23	0.66	0.58	0.70	0.46	0.85	0.55	0.99
	*S24–7*	0.23	0.83	0.89	0.56	0.95	0.42	0.96	0.21
	*Selenomonas*	0.75	0.01*	0.65	0.03*	0.07^a^	0.70	0.39	0.05*
	Shannon	0.25	0.04*	0.13	0.02*	0.33	0.58	0.77	0.41

^a^Variables not statistically significant but present in the model

Among other major genera, *Megasphaera* was strongly positively correlated with tPC1 and pPC7 (FDR *P* = 2.0E-4), which were negatively enriched for host immune pathways such as IL-17 and interferon pathways (Fig. 4b) and associated with reduced expression of pro-inflammatory markers such as C-C motif chemokine 20 and interleukin-36 (Fig. 4c, Additional file 4). Therefore, increased abundance of *Megasphaera* could be associated with reduced airway inflammatory responses. In comparison, other major genera such as *Streptococcus* and *Veillonella* were associated with relatively little host response.

Within stable samples only, the *Haemophilus*-tPC2-pPC3 associations persisted, while *Moraxella* was not associated with any host PCs (Additional file 1: Figure S8, Tables 2-3). Within stable, *Streptococcus* showed a significantly negative correlation with tPC6 (FDR *P* = 3E-3, Table 2, Additional file 3), in which several phagocytosis and neutrophil migration pathways were most negatively enriched. Thus, increased *Streptococcus* could be associated with greater expression of these pathways at stable. Furthermore, an unknown genus in S24–7 family had significant positive correlations with tPC9 at stable (FDR *P* = 2E-3, Table 2, Additional file 3), in which IL-12, IL-23 and T-cell differentiation pathways were most negatively enriched. This genus, despite its low abundance, could be associated with reduced inflammatory response at stable.

## Discussion

Here we present the first comprehensive study characterizing airway host-microbiome interactions in COPD integrating lung microbiome and host multi-omics datasets both in stable state and during exacerbations. The systems biology approach revealed a significant airway host-microbiome interplay associated with COPD inflammation and exacerbations. Among all major genera, *Haemophilus* and *Moraxella* were most strongly associated with host gene expression profiles, particularly immunity and inflammation, suggesting the two genera as key players in airway host-bacterial crosstalk in COPD.

Importantly, our results revealed different timing of host responses to these two genera. While *Haemophilus* was associated with host responses both in stable state and during exacerbations, the associations for *Moraxella* were primarily related to exacerbations. This is consistent with a previous study [42] and highlights the role of *Haemophilus* as a stable airway colonizer and *Moraxella* as an exacerbation-related opportunistic pathogen in COPD. Furthermore, the *Haemophilus*-associated immune responses were correlated with the degree of neutrophilic inflammation, underscoring the interactions between bacterial presence, host immune responses and cellular inflammation. This suggests that chronic airway inflammation in some COPD patients may not respond to anti-inflammatory therapies alone [43] unless the underlying bacterial infection driving the abnormal immune response is addressed.

To achieve statistical power for a genome-wide analysis associating microbiome and host multi-omics datasets in a relatively small sample set, we performed dimensionality reduction on host data and used multivariate modeling to identify significant associations between microbiome composition and overall patterns of host gene expression. Similar approaches were employed by Morgan et al. in associating gut microbiome with host transcriptome in inflammatory bowel disease patients [29]. The strong correlations of *Haemophilus* and *Moraxella* with host immune and inflammation-related tPCs and pPCs highlight the positive links between the two genera and host immune responses that predominated airway host-microbiome interactions. The presence of lipopolysaccharide-induced bronchial epithelial receptors among the top loadings demonstrates that our approach can recapitulate an active host-bacterial crosstalk in COPD. While both *Haemophilus* and *Moraxella* were positively associated with T-cell induced pro-inflammatory signaling, the interferon signaling was more strongly linked to *Moraxella* than *Haemophilus*. This is consistent with one previous study showing that *M. catarrhalis* but not *H. influenzae* induced interferon-beta expression in bronchial epithelial cells [44] and aligns with the different pathogenicity profiles between the two pathogens [45]. Differential involvement of viral co-infection could be another important factor [46]. We have not fully characterized the sputum viral load of this cohort due to limited sputum available. Additional viral load data is key to further resolving this question.

Our multivariate analysis showed that *Megasphaera* and an unknown genus in S24–7 family were associated with reduced expression of host inflammatory pathways and therefore could potentially reverse airway inflammation (i.e. interferon, IL-12 pathway) induced by *Haemophilus* and *Moraxella*. Furthermore, *Megasphaera* was negatively correlated with sputum neutrophil counts. *Megasphaera* is a known member of human lung microbiome [39] and has beneficial effects on the host through short chain fatty acids (SCFAs) production [47]. In the lung microenvironment, bacterial SCFAs were shown to inhibit cytokine production and inflammation after LPS stimulation of macrophages [48]. Trompette et al. also showed that bacterial SCFAs reduce neutrophil recruitment to the airways and protect against influenza virus infection in mice, suggesting that it has anti-inflammatory effects [49]. Cait et al. demonstrated that diet-derived SCFAs ameliorate allergic inflammation in mice, suggesting its anti-inflammatory effects in the lung [50]. One study on oropharyngeal microbiome of H7N9-infected patients showed that *Megasphaera* increased in patients without secondary bacterial infection, suggesting its potential role in preventing colonization of respiratory pathogens [51]. Further validation on the identity and prevalence of these genera is warranted to explore their functions in the COPD lung.

Our study provides novel insights on the impact of smoking on the lung microbiome, although individual subgroups had small sample sizes and the results need further confirmation in larger cohorts. Our results suggest that the effect of current smoking on the lung microbiome differs between healthy subjects and COPD patients. In healthy subjects, a significantly increased *Haemophilus* was observed in smokers versus non-smokers, suggesting that smoking could be a risk factor for airway dysbiosis in healthy populations. In COPD patients, a significantly increased *Haemophilus* was observed in ex-smokers versus current smokers. The greater dysbiosis in COPD ex-smokers was further associated with their greater airway inflammatory states, as evident by significantly higher expression of sputum pro-inflammatory markers. Our findings further support the view that smoking likely had resulted in an irreversible airway inflammation in COPD, which persisted despite smoking cessation [52].

We observed significant increase of *Moraxella* in stable COPD patients versus healthy subjects, and in COPD exacerbations versus stable, in agreement with previous observations [5, 8, 9, 36]. The reversal trends of microbiome diversity and composition prior and post exacerbations further support the lung microbiome dysbiosis during exacerbations. Increased *Haemophilus* and *Moraxella* were found in stable ICS versus non-ICS exposed patients, consistent with earlier observations [6, 9], with the caveat being the small sample size of non-ICS users. At stable, the microbiome was comparable between frequent and infrequent exacerbators, suggesting that the baseline microbiome does not effectively predict exacerbation frequency. Identifying markers that predict the exacerbation frequency is of great importance for COPD management [53]. Differences in baseline respiratory microbiota composition were hypothesized to explain the different exacerbation frequency in COPD patients [13]. However, neither this study nor earlier reports support this hypothesis [54, 11]. Instead, previous longitudinal studies showed that there is an association between temporal variability of the airway microbiome and patient exacerbation frequency [11, 12], suggesting that the frequent exacerbator phenotype might be more relevant to the de-stabilization of the microbiome over time but not the microbiome composition at baseline per se. We observed no significant association between microbiome or sputum cell count changes with CAT score, FEV1 or FVC, which suggests that different patient inflammatory profiles (i.e. neutrophilic or eosinophilic inflammation) and their associated airway microbiome changes are likely independent of disease severity and cannot be distinguished clinically [55].

There are several caveats to our study. First, the sample size was relatively small particularly for the subgroup analysis, and the longitudinal profiling was limited due to the limited amount of sputum produced in some visits and the technical difficulty in extracting sufficient material from sputum for the various aspects of downstream experiments (i.e. microbiome, transcriptome, proteome, cell counting). We performed a power estimation to ensure adequate statistical sensitivity could be achieved after dimensionality reduction. Nevertheless, the associations observed in our study need to be validated in larger independent patient cohorts. Second, host transcriptome and proteome were not profiled for healthy subjects, which is important to understand to what extent the observed host-microbiome associations are disease specific. Our study provides a method for profiling airway host-microbiome interactions that should catalyze future efforts on characterizing lung microbiome and host multi-omics in larger healthy and disease populations.

## Conclusions

To our knowledge, this is the first study that depicts airway host-microbiome interactions in COPD and highlights the differential role of *Haemophilus* and *Moraxella* in terms of host interactions. Our study provides support for novel therapies targeting both genera and their associated host pathways to overcome the abnormal immune response in COPD.

## Additional files

Additional file 1: Supplementary figures and tables (DOCX 856 kb)

Additional file 2: a. MetaCore pathways significantly enriched for differential expressed genes (DEGs) between stable and exacerbations (FDR *P* ≤ 0.01). b. Significantly differentially expressed proteins between sputum samples of COPD ex-smokers and current smokers (Wilcoxon, FDR *P* ≤ 0.05). (XLSX 68 kb)

Additional file 3: Cross associations among microbiome (9 microbiome genera and Shannon diversity), host transcriptome (9 tPCs) and proteome (8 pPCs) data both across all samples and within stable samples in the multivariate analysis. FDR *P*-values are indicated in the table. Significant positive correlations are highlighted in red. Significant negative correlations are highlighted in blue. (XLSX 15 kb)

Additional file 4: a. Top 25 loadings by magnitude for each tPC. b. Top 25 loadings by magnitude for each pPC. c. Top 10 positively and negatively enriched pathways and their enrichment scores as shown in the heatmap in Fig. 4b. d. Top 25 significantly enriched positive and negative pathways (FDR *P* < 0.01) for the loadings of tPC1–9. (XLSX 103 kb)

Additional file 5: Spearman correlation of individual genes in the top two pathways. **a.** tPC2 and **b.** tPC4 with *Haemophilus* and *Moraxella* in both microbiome and qPCR datasets. (XLSX 32 kb)

## Abbreviations

FDR: False discovery rate
GLMM: Generalized linear mixed model
GSEA: Gene set enrichment analysis
ICS: Inhaled corticosteroid
LPS: Lipopolysaccharide
OTUs: Operational taxonomic units
PCA: Principal component analysis
QoL: Quality of life
qPCR: Quantitative polymerase chain reaction
SCFA: Short chain fatty acid

## References

[1] Ball P (1995). Epidemiology and treatment of chronic bronchitis and its exacerbations. Chest.

[2] Miravitlles M, Espinosa C, Fernandez-Laso E, Martos JA, Maldonado JA, Gallego M (1999). Relationship between bacterial flora in sputum and functional impairment in patients with acute exacerbations of COPD. Study Group of Bacterial Infection in COPD. Chest.

[3] Monso E, Ruiz J, Rosell A, Manterola J, Fiz J, Morera J (1995). Bacterial infection in chronic obstructive pulmonary disease. A study of stable and exacerbated outpatients using the protected specimen brush. Am J Respir Crit Care Med.

[4] Soler N, Torres A, Ewig S, Gonzalez J, Celis R, El-Ebiary M (1998). Bronchial microbial patterns in severe exacerbations of chronic obstructive pulmonary disease (COPD) requiring mechanical ventilation. Am J Respir Crit Care Med.

[5] Hilty M, Burke C, Pedro H, Cardenas P, Bush A, Bossley C (2010). Disordered microbial communities in asthmatic airways. PLoS One.

[6] Pragman AA, Kim HB, Reilly CS, Wendt C, Isaacson RE (2012). The lung microbiome in moderate and severe chronic obstructive pulmonary disease. PLoS One.

[7] Sze MA, Dimitriu PA, Hayashi S, Elliott WM, McDonough JE, Gosselink JV (2012). The lung tissue microbiome in chronic obstructive pulmonary disease. Am J Respir Crit Care Med.

[8] Huang YJ, Sethi S, Murphy T, Nariya S, Boushey HA, Lynch SV (2014). Airway microbiome dynamics in exacerbations of chronic obstructive pulmonary disease. J Clin Microbiol.

[9] Wang Z, Bafadhel M, Haldar K, Spivak A, Mayhew D, Miller BE (2016). Lung microbiome dynamics in chronic obstructive pulmonary disease exacerbations. The European respiratory journal.

[10] Millares L, Ferrari R, Gallego M, Garcia-Nunez M, Perez-Brocal V, Espasa M (2014). Bronchial microbiome of severe COPD patients colonised by Pseudomonas aeruginosa. European journal of clinical microbiology & infectious diseases : official publication of the European Society of Clinical Microbiology.

[11] Wang Zhang, Singh Richa, Miller Bruce E, Tal-Singer Ruth, Van Horn Stephanie, Tomsho Lynn, Mackay Alexander, Allinson James P, Webb Adam J, Brookes Anthony J, George Leena M, Barker Bethan, Kolsum Umme, Donnelly Louise E, Belchamber Kylie, Barnes Peter J, Singh Dave, Brightling Christopher E, Donaldson Gavin C, Wedzicha Jadwiga A, Brown James R (2017). Sputum microbiome temporal variability and dysbiosis in chronic obstructive pulmonary disease exacerbations: an analysis of the COPDMAP study. Thorax.

[12] Mayhew David, Devos Nathalie, Lambert Christophe, Brown James R, Clarke Stuart C, Kim Viktoriya L, Magid-Slav Michal, Miller Bruce E, Ostridge Kristoffer K, Patel Ruchi, Sathe Ganesh, Simola Daniel F, Staples Karl J, Sung Ruby, Tal-Singer Ruth, Tuck Andrew C, Van Horn Stephanie, Weynants Vincent, Williams Nicholas P, Devaster Jeanne-Marie, Wilkinson Tom M A (2018). Longitudinal profiling of the lung microbiome in the AERIS study demonstrates repeatability of bacterial and eosinophilic COPD exacerbations. Thorax.

[13] Dickson RP, Martinez FJ, Huffnagle GB (2014). The role of the microbiome in exacerbations of chronic lung diseases. Lancet.

[14] Sze MA, Dimitriu PA, Suzuki M, McDonough JE, Campbell JD, Brothers JF (2015). Host response to the lung microbiome in chronic obstructive pulmonary disease. Am J Respir Crit Care Med.

[15] Molyneaux PL, Willis-Owen SAG, Cox MJ, James P, Cowman S, Loebinger M (2017). Host-microbial interactions in idiopathic pulmonary fibrosis. Am J Respir Crit Care Med.

[16] General Assembly of the World Medical A (2014). World medical association declaration of Helsinki: ethical principles for medical research involving human subjects. J Am Coll Dent.

[17] International Conference on (2001). Harmonisation of technical requirements for registration of pharmaceuticals for human u. ICH harmonized tripartite guideline: guideline for good clinical practice. J Postgrad Med.

[18] Anthonisen NR, Manfreda J, Warren CP, Hershfield ES, Harding GK, Nelson NA (1987). Antibiotic therapy in exacerbations of chronic obstructive pulmonary disease. Ann Intern Med.

[19] Rodriguez-Roisin R (2000). Toward a consensus definition for COPD exacerbations. Chest.

[20] Bafadhel M, McCormick M, Saha S, McKenna S, Shelley M, Hargadon B (2012). Profiling of sputum inflammatory mediators in asthma and chronic obstructive pulmonary disease. Respiration.

[21] Caporaso JG, Kuczynski J, Stombaugh J, Bittinger K, Bushman FD, Costello EK (2010). QIIME allows analysis of high-throughput community sequencing data. Nat Methods.

[22] Salter SJ, Cox MJ, Turek EM, Calus ST, Cookson WO, Moffatt MF (2014). Reagent and laboratory contamination can critically impact sequence-based microbiome analyses. BMC Biol.

[23] Wang X, Lin Y, Song C, Sibille E, Tseng GC (2012). Detecting disease-associated genes with confounding variable adjustment and the impact on genomic meta-analysis: with application to major depressive disorder. BMC Bioinf.

[24] Menni C, Kiddle SJ, Mangino M, Vinuela A, Psatha M, Steves C (2015). Circulating proteomic signatures of chronological age. J Gerontol A Biol Sci Med Sci.

[25] Mehan MR, Williams SA, Siegfried JM, Bigbee WL, Weissfeld JL, Wilson DO (2014). Validation of a blood protein signature for non-small cell lung cancer. Clin Proteomics.

[26] Hathout Y, Brody E, Clemens PR, Cripe L, DeLisle RK, Furlong P (2015). Large-scale serum protein biomarker discovery in Duchenne muscular dystrophy. Proc Natl Acad Sci U S A.

[27] Robinson MD, McCarthy DJ, Smyth GK (2010). edgeR: a Bioconductor package for differential expression analysis of digital gene expression data. Bioinformatics.

[28] Ritchie ME, Phipson B, Wu D, Hu Y, Law CW, Shi W (2015). Limma powers differential expression analyses for RNA-sequencing and microarray studies. Nucleic Acids Res.

[29] Morgan XC, Kabakchiev B, Waldron L, Tyler AD, Tickle TL, Milgrom R (2015). Associations between host gene expression, the mucosal microbiome, and clinical outcome in the pelvic pouch of patients with inflammatory bowel disease. Genome Biol.

[30] Bates DMM, Bolker B, Walker S (2015). Fitting linear mixed-effects models using lme4. J Stat Softw.

[31] BPaCR KA (2014). lmerTest: tests in linear mixed effects models.

[32] Brindefalk B, Ettema TJ, Viklund J, Thollesson M, Andersson SG (2011). A phylometagenomic exploration of oceanic alphaproteobacteria reveals mitochondrial relatives unrelated to the SAR11 clade. PLoS One.

[33] Subramanian A, Tamayo P, Mootha VK, Mukherjee S, Ebert BL, Gillette MA (2005). Gene set enrichment analysis: a knowledge-based approach for interpreting genome-wide expression profiles. Proc Natl Acad Sci U S A.

[34] Reich M, Liefeld T, Gould J, Lerner J, Tamayo P, Mesirov JP (2006). GenePattern 2.0. Nat Genet.

[35] Benjamini Y, Hochberg Y (1995). Controlling the false discovery rate – a practical and powerful approach to multiple testing. J R Stat Soc Ser B Methodol.

[36] Molyneaux PL, Mallia P, Cox MJ, Footitt J, Willis-Owen SA, Homola D (2013). Outgrowth of the bacterial airway microbiome after rhinovirus exacerbation of chronic obstructive pulmonary disease. Am J Respir Crit Care Med.

[37] Erb-Downward JR, Thompson DL, Han MK, Freeman CM, McCloskey L, Schmidt LA (2011). Analysis of the lung microbiome in the "healthy" smoker and in COPD. PLoS One.

[38] Huang YJ, Kim E, Cox MJ, Brodie EL, Brown R, Wiener-Kronish JP (2010). A persistent and diverse airway microbiota present during chronic obstructive pulmonary disease exacerbations. Omics.

[39] Zakharkina T, Heinzel E, Koczulla RA, Greulich T, Rentz K, Pauling JK (2013). Analysis of the airway microbiota of healthy individuals and patients with chronic obstructive pulmonary disease by T-RFLP and clone sequencing. PLoS One.

[40] Mofarrahi M, Nouh T, Qureshi S, Guillot L, Mayaki D, Hussain SN (2008). Regulation of angiopoietin expression by bacterial lipopolysaccharide. Am J Physiol Lung Cell Mol Physiol.

[41] Ivanov AI, Romanovsky AA (2006). Putative dual role of ephrin-Eph receptor interactions in inflammation. IUBMB Life.

[42] Barker BL, Haldar K, Patel H, Pavord ID, Barer MR, Brightling CE (2015). Association between pathogens detected using quantitative polymerase chain reaction with airway inflammation in COPD at stable state and exacerbations. Chest.

[43] King PT (2015). Inflammation in chronic obstructive pulmonary disease and its role in cardiovascular disease and lung cancer. Clin Transl Med.

[44] Klaile E, Klassert TE, Scheffrahn I, Muller MM, Heinrich A, Heyl KA (2013). Carcinoembryonic antigen (CEA)-related cell adhesion molecules are co-expressed in the human lung and their expression can be modulated in bronchial epithelial cells by non-typable Haemophilus influenzae, Moraxella catarrhalis, TLR3, and type I and II interferons. Respir Res.

[45] Sethi S, Murphy TF (2001). Bacterial infection in chronic obstructive pulmonary disease in 2000: a state-of-the-art review. Clin Microbiol Rev.

[46] DeMuri GP, Gern JE, Eickhoff JC, Lynch SV, Wald ER (2017). Dynamics of bacterial colonization with Streptococcus pneumoniae, Haemophilus influenzae and Moraxella catarrhalis during symptomatic and asymptomatic viral upper respiratory infection. Clinical infectious diseases : an official publication of the Infectious Diseases Society of America.

[47] Shetty SA, Marathe NP, Lanjekar V, Ranade D, Shouche YS (2013). Comparative genome analysis of Megasphaera sp. reveals niche specialization and its potential role in the human gut. PLoS One.

[48] Chang PV, Hao L, Offermanns S, Medzhitov R (2014). The microbial metabolite butyrate regulates intestinal macrophage function via histone deacetylase inhibition. Proc Natl Acad Sci U S A.

[49] Trompette A, Gollwitzer ES, Pattaroni C, Lopez-Mejia IC, Riva E, Pernot J (2018). Dietary Fiber confers protection against flu by shaping Ly6c(−) patrolling monocyte hematopoiesis and CD8(+) T cell metabolism. Immunity.

[50] Cait A, Hughes MR, Antignano F, Cait J, Dimitriu PA, Maas KR (2018). Microbiome-driven allergic lung inflammation is ameliorated by short-chain fatty acids. Mucosal Immunol.

[51] Lu HF, Li A, Zhang T, Ren ZG, He KX, Zhang H (2017). Disordered oropharyngeal microbial communities in H7N9 patients with or without secondary bacterial lung infection. Emerg Microbes Infect.

[52] Laniado-Laborin R (2009). Smoking and chronic obstructive pulmonary disease (COPD). Parallel epidemics of the 21 century. Int J Environ Res Public Health.

[53] Wedzicha JA, Brill SE, Allinson JP, Donaldson GC (2013). Mechanisms and impact of the frequent exacerbator phenotype in chronic obstructive pulmonary disease. BMC Med.

[54] Wang Z, Bafadhel M, Haldar K, Spivak A, Mayhew D, Miller BE (2016). Lung microbiome dynamics in COPD exacerbations. Eur Respir J.

[55] Bafadhel M, McKenna S, Terry S, Mistry V, Reid C, Haldar P (2011). Acute exacerbations of chronic obstructive pulmonary disease: identification of biologic clusters and their biomarkers. Am J Respir Crit Care Med.

