# Supplementary material for: Airway host-microbiome interactions in chronic obstructive pulmonary disease
Source: Respir Res. 2019 Jun 6;20:113. doi: 10.1186/s12931-019-1085-z (PMC6555748; doi:10.1186/s12931-019-1085-z)
Supplement: Supplementary file 1 — Supplementary figures and tables (DOCX 856 kb) [file 12931_2019_1085_MOESM1_ESM.docx]

**Airway host-microbiome interactions in chronic obstructive pulmonary disease**

Additional Material

Zhang Wang^1.2&^, Barbara Maschera^3^, Simon Lea^4^, Umme Kolsum^4^, David Michalovich^3^, Stephanie Van Horn^5^, Christopher Traini^5^, James R. Brown^1*^, Edith M. Hessel^3#^, Dave Singh^4#^

^1^ Computational Biology, Human Genetics, Research and Development (R&D), GlaxoSmithKline (GSK), Collegeville, PA 19426, USA

^2^ School of Life Sciences, South China Normal University, Guangzhou, 510631, P.R. China.

^3^ Refractory Respiratory Inflammation, Discovery Performance Unit, Respiratory Therapy Area, R&D, GSK, Stevenage, SG1 2NY, UK

^4^ University of Manchester and University Hospital of South Manchester, Manchester, M23 9QZ, UK

^5^ Functional Genomics, Medicinal Science & Technology, R&D, GSK, Collegeville, PA 19426, USA

^#^ Co-senior authors
^&^ Current address

*** Correspondence to:** James R. Brown ([James.R.Brown@gsk.com](mailto:James.R.Brown@gsk.com))

1250 S. Collegeville Road, Collegeville, Pennsylvania, 19426-0989, United States


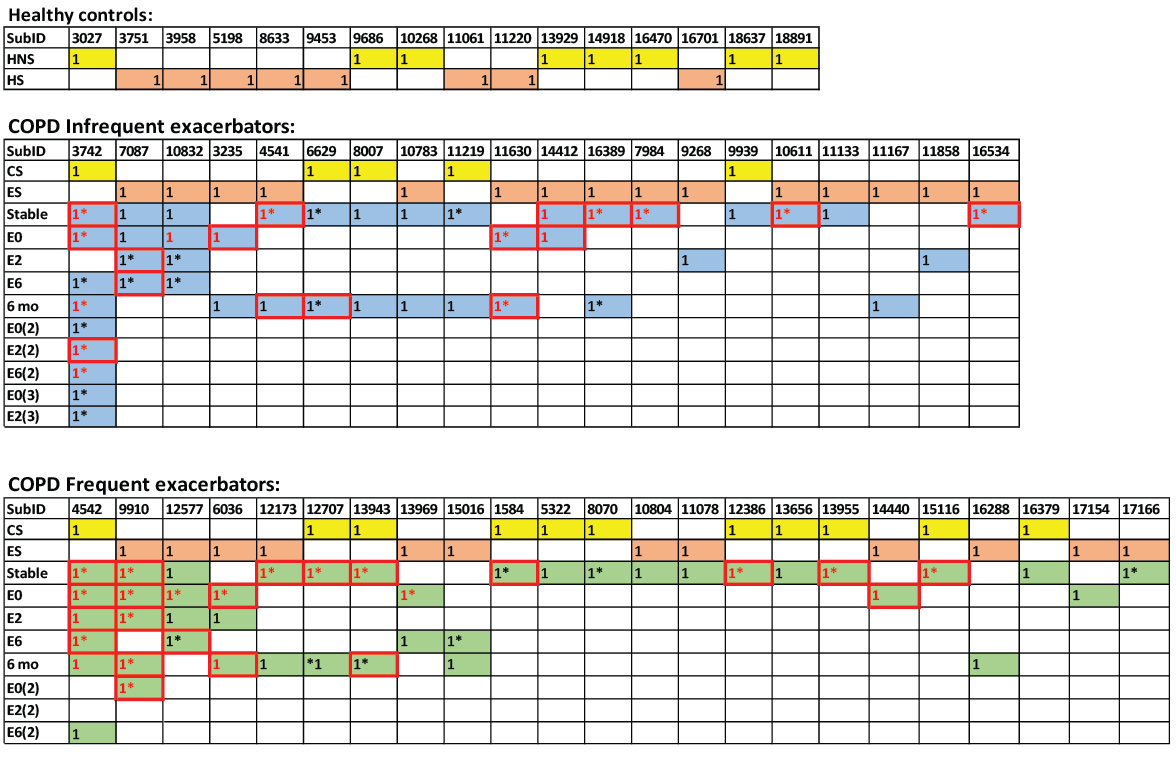


**Figure S1.** Time points of sputum microbiome sampling for healthy and COPD patients in this study. Three patients (3742, 4542 and 9910) with recurrent exacerbations had multiple series of sputum sample collection. HNS: healthy non-smokers, HS: healthy smokers, CS: COPD current smokers, ES: COPD ex-smokers, E0: exacerbations, E2: two week post-exacerbations, E6: six week post-exacerbations, 6 mo: six months from first stable visit. The samples with host transcriptome data are highlighted in red borders. The samples with Somalogic proteomic data are highlighted in red text. The samples with sputum cell count data are indicated in asterisks. The missing samples are mostly due to patients unable to produce sufficient amount of sputum for downstream experiments.


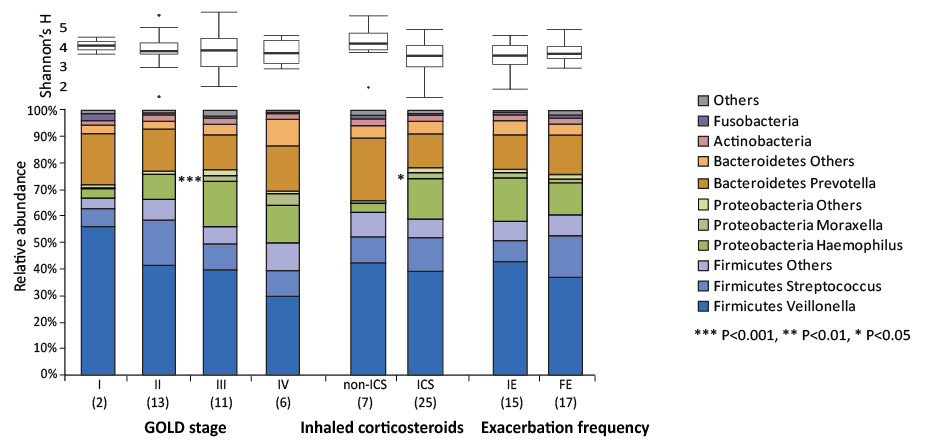


**Figure S2.** Shannon diversity and relative abundance of major bacterial taxa in stable COPD GOLD I-IV patients, ICS and non-ICS exposed patients, and frequent and infrequent exacerbators. Significantly differentially represented bacterial taxa were identified using edgeR([1](#_ENREF_1)). For GOLD status, statistical analysis was performed on each adjacent two groups. E0: COPD exacerbations, IE: infrequent exacerbators, FE: frequent exacerbators. *** FDR *P* < 0.001, ** FDR *P* < 0.01, * FDR *P* < 0.05.


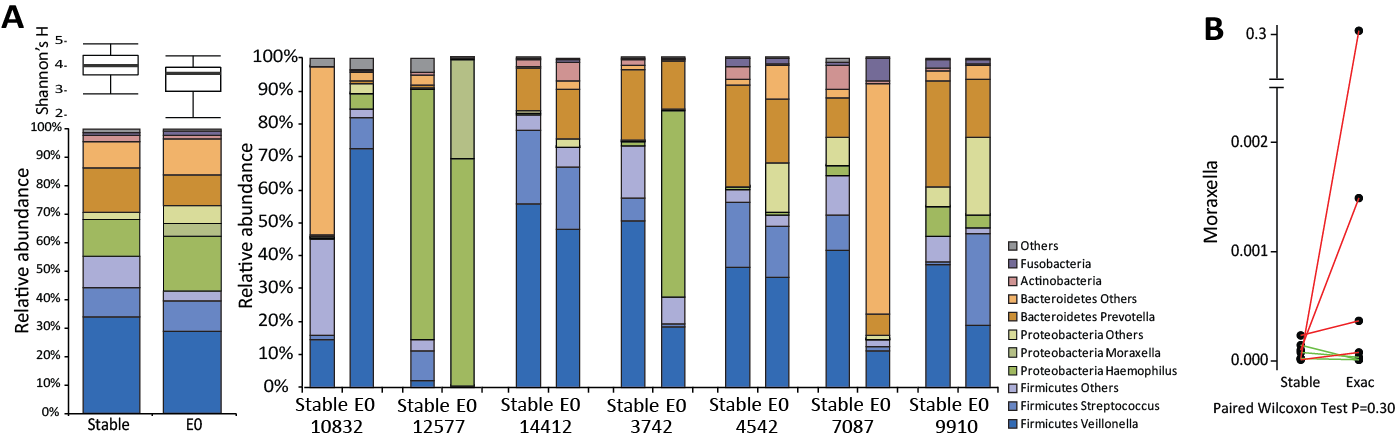


**Figure S3. a.** Sputum microbiome profiles of paired stable and exacerbation samples from the same patients. **b.** A non-significant increase of *Moraxella* in paired exacerbation versus stable samples. E0: exacerbations.


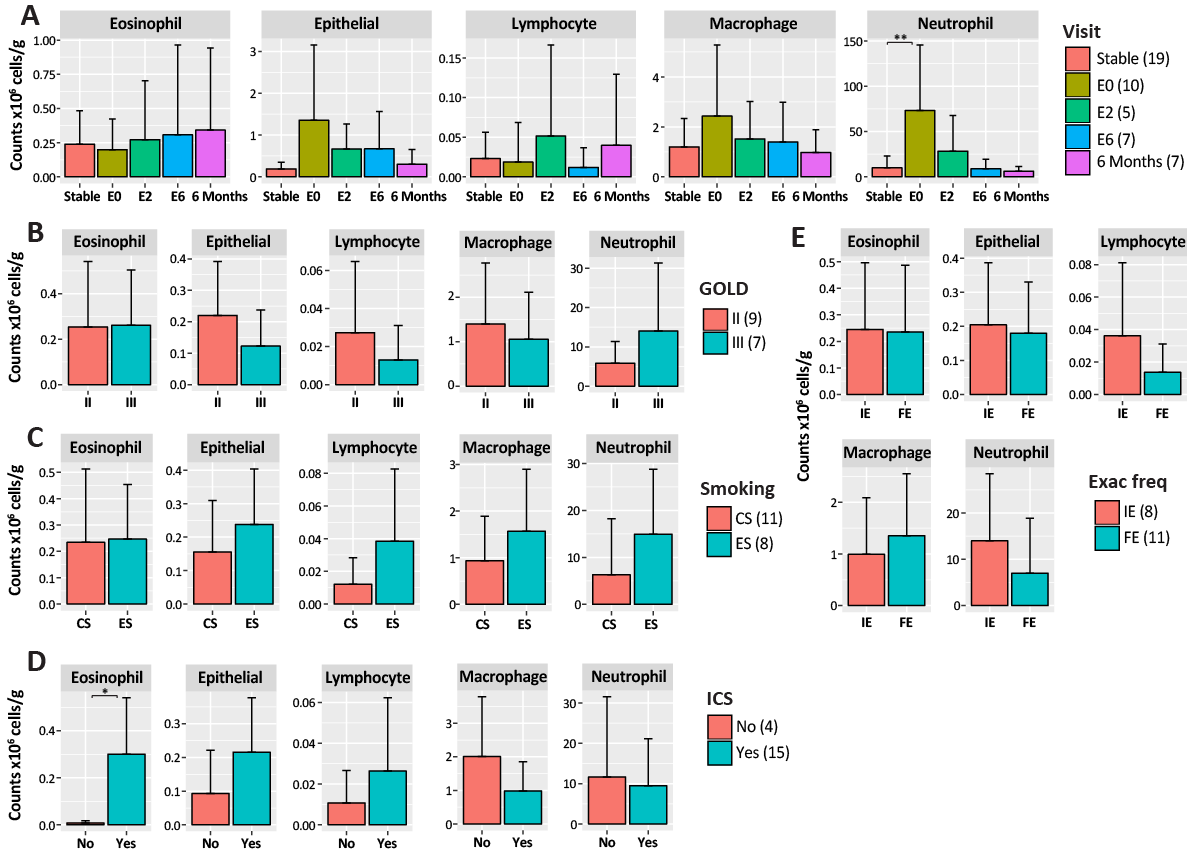


**Figure S4.** Barplots showing the mean and standard deviation of sputum absolute immune cell counts (eosinophil, epithelial, lymphocyte, macrophage and neutrophil cells) in COPD patients **a.** at different visits, and in stable state of **b.** GOLD II and III patients, **c.** COPD current smokers (CS) and ex-smokers (ES), and **d.** ICS and non-ICS exposed patients and **e.** infrequent (IE) and frequent (FE) exacerbators. *** FDR *P* < 0.001, ** FDR *P* < 0.01, * FDR *P* < 0.05.


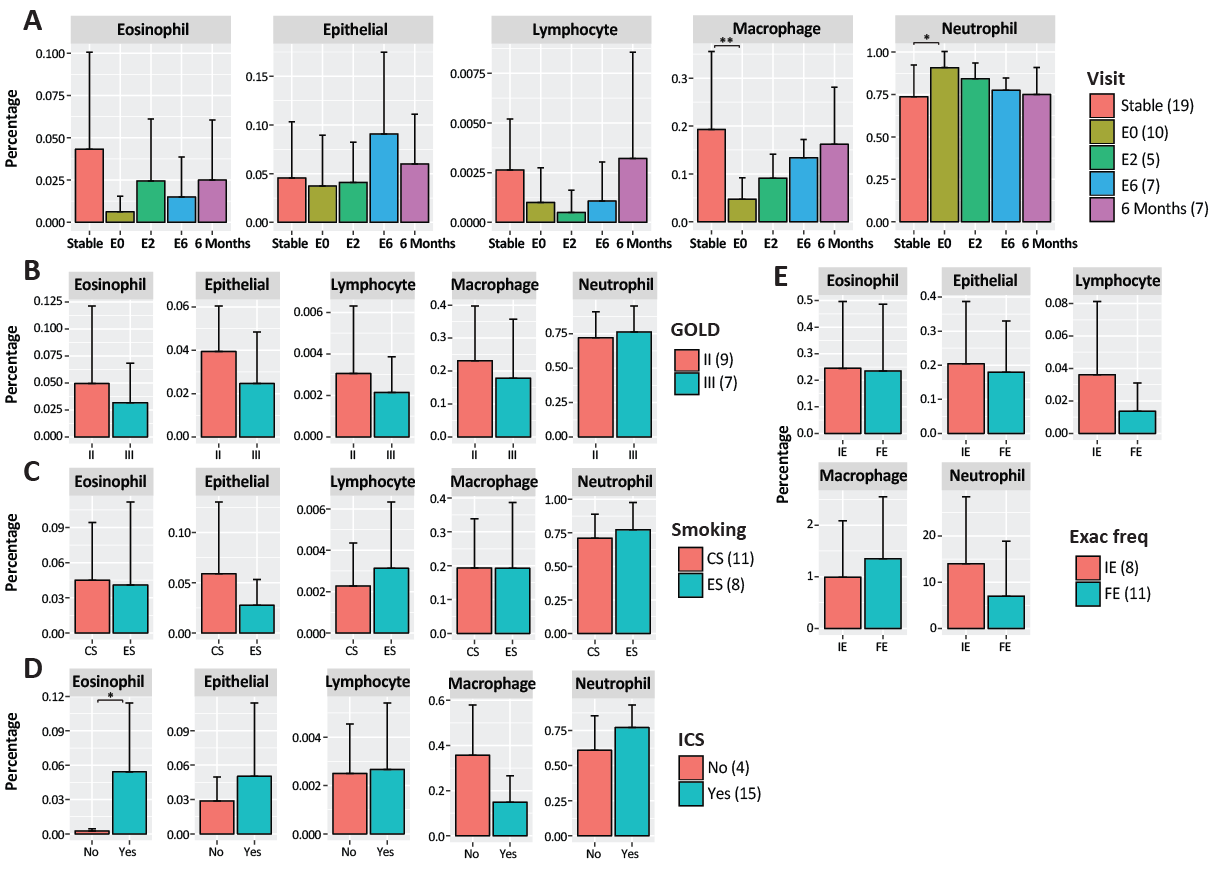


**Figure S5.** Barplots showing the mean and standard deviation of sputum cell percentage (eosinophil, epithelial, lymphocyte, macrophage and neutrophil cells) in COPD patients. **a.** At different visits. **b.** Stable state of GOLD II and III patients. **c.** COPD current smokers (CS) and ex-smokers (ES). **d.** ICS and non-ICS exposed patients. **e.** infrequent (IE) and frequent (FE) exacerbators. *** FDR *P* < 0.001, ** FDR *P* < 0.01, * FDR *P* < 0.05.


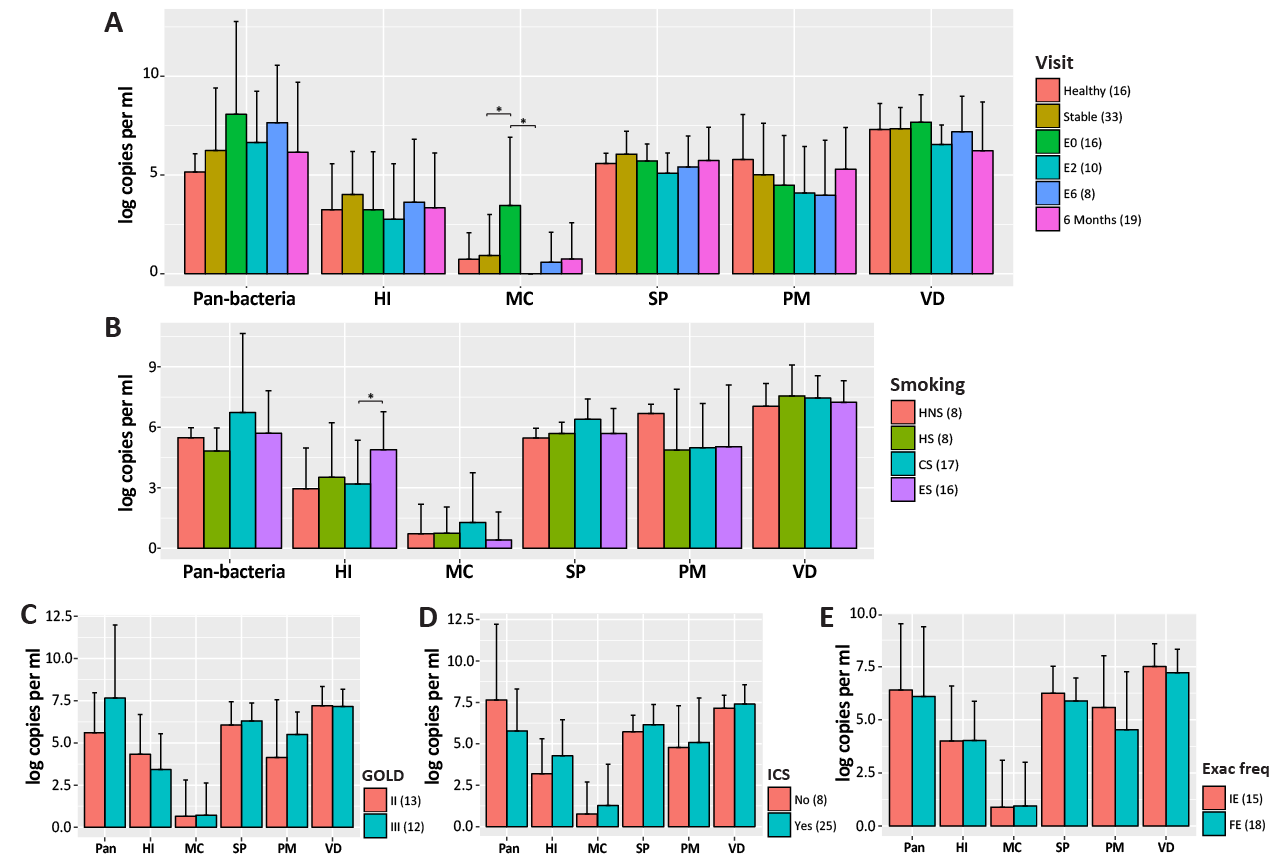


**Figure S6.** Barplots showing the mean and standard deviation of quantitative PCR total bacterial load. **a.** In healthy subjects and COPD patients at different visits. **b.** In healthy smokers (HS) and non-smokers (HNS), and in stable state of COPD ex-smokers (ES) and current smokers (CS). **c.** In stable state of COPD GOLD status II and III patients. **d.** In stable state of ICS and non-ICS exposed patients. **e.** In stable state of infrequent (IE) and frequent (FE) exacerbators. HI: *Haemophilus influenzae*, MC: *Moraxella catarrhalis*, SP: *Streptococcus pneumoniae*, PM: *Prevotella melaninogenica*, VD: *Veillonella dispar*. *** FDR *P* < 0.001, ** FDR *P* < 0.01, * FDR *P* < 0.05.


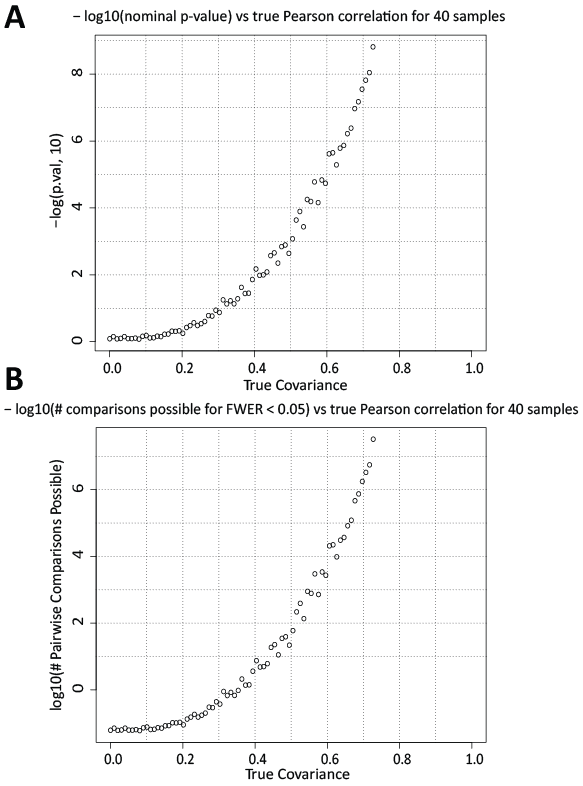


**Figure S7.** Statistical power estimates. **a.** The 80th percentile of raw *P*-values of Spearman correlation test, as a function of true covariance between the simulated variables. Variables are standard normal distributed, so covariance equals Pearson product moment. **b.** Number of tests possible to retain 80% power and alpha equal to 0.05, using Bonferroni correction. Variables are standard normal distributed, so covariance equals Pearson product moment.


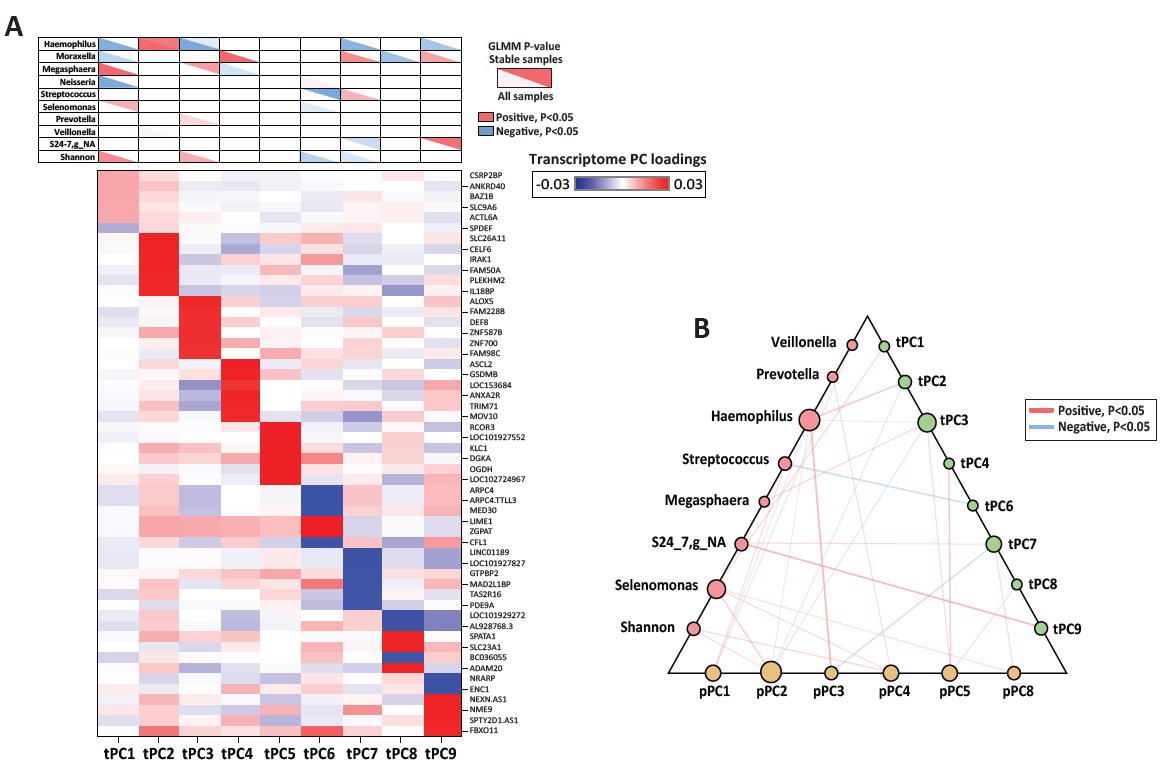


**Figure S8.** Microbiome-transcriptome multivariate analysis showed strongest association of *Haemophilus* and *Moraxella* with host transcriptome. **a.** Shown in the top table are associations of the 9 most abundant genera and Shannon diversity with tPCs both across all samples (bottom left of the table entry) and within stable samples (top right of the table entry) in the GLMM. Significant associations (FDR *P* ≤ 0.05) are colored by direction. The strength of the color corresponds to the *P*-value significance. Shown in the bottom heatmap are the top loadings of tPCs. For each tPC, the top 6 genes by magnitude of loadings are shown. **b.** Host-microbiome interaction network illustrating significant associations among the 9 most abundant microbiome genera and Shannon diversity, tPCs and pPCs in GLM within COPD stable state. Each edge indicates a significant association (FDR *P* ≤ 0.05) colored by direction. The edge weight corresponds to the significance of the *P*-value. The size of the node is proportional to the number of significant associations involving the node.


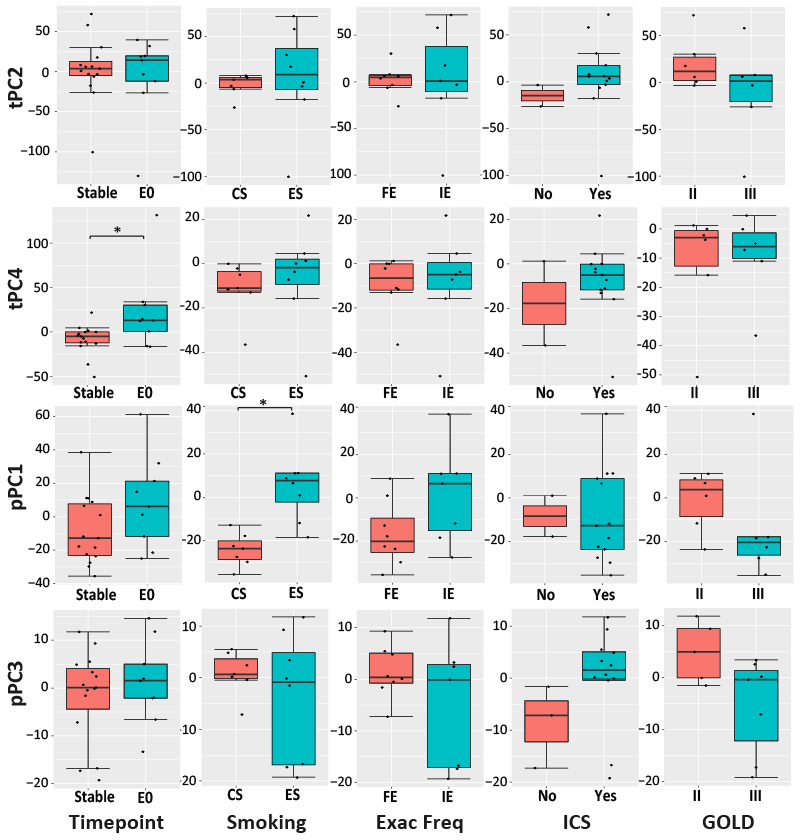


**Figure S9.** Changes of tPC2, tPC4, pPC1 and pPC3 between stable and exacerbation (E0) samples, and between stable samples of COPD current smokers (CS) and ex-smokers (ES), GOLD II and III patients, ICS and non-ICS exposed patients, and infrequent (IE) and frequent (FE) exacerbators. *** FDR *P* < 0.001, ** FDR *P* < 0.01, * FDR *P* < 0.05.

**Table S1.** Major longitudinal clinical features of COPD subjects in this study.

| **Longitudinal clinical features** | **Visit** |  | ***P*-value*** |
| --- | --- | --- | --- |
|  | **Stable (N=32)** | **E0 (N=16)** |  |
| Pre FEV_1_ (L)^†^ | 1.1 (0.5) | NA^¶^ | NA |
| Pre FEV_1_% | 42.3 (16.8) | 37.3 (11.0) | 0.27 |
| Pre FVC (L) | 2.8 (0.9) | NA | NA |
| Pre FVC% | 81.2 (20.0) | NA | NA |
| Pre FEV_1_/FVC ratio | 0.4 (0.1) | NA | NA |
| Post FEV_1_ (L) | 1.2 (0.6) | 1.0 (0.4) | 0.44 |
| Post FEV_1_% | 51.2 (18.0) | 42.4 (13.2) | 0.08 |
| Post FVC (L) | 3.3 (1.0) | 3.1 (0.8) | 0.54 |
| Post FVC% | 95.9 (21.3) | 92.9 (19.0) | 0.67 |
| Post FEV_1_/FVC ratio | 0.4 (0.1) | 0.4 (0.1) | 0.16 |
| Sputum neutrophil count (x10^6^ cells/g) | 9.94 (13.13) | 91.37 (79.10) | 0.02 |
| Sputum macrophage count (x10^6^ cells/g) | 1.20 (1.14) | 3.07 (3.23) | 0.22 |
| Sputum eosinophil count (x10^6^ cells/g)^#^ | 0.25 (0.44) | 0.16 (0.45) | 0.58^‡^ |
| Sputum lymphocyte count (x10^6^ cells/g) | 0.02 (0.03) | 0.02 (0.06) | 0.8 |
| Sputum epithelial count (x10^6^ cells/g) | 0.19 (0.16) | 1.73 (2.08) | 0.07 |
| Sputum neutrophil percentage (%)^#^ | 73.68 (18.69) | 92.39 (8.03) | 0.01^‡^ |
| Sputum macrophage percentage (%)^#^ | 19.26 (16.29) | 4.68 (5.23) | 0.01^‡^ |
| Sputum eosinophil percentage (%)^#^ | 2.00 (7.00) | 0.25 (0.50) | 0.05^‡^ |
| Sputum lymphocyte percentage (%)^#^ | 0.26 (0.26) | 0.07 (0.12) | 0.08^‡^ |
| Sputum epithelial percentage (%)^#^ | 4.57 (5.78) | 2.50 (2.79) | 0.39^‡^ |

^†^ Continuous data present as mean (SD) unless stated below.

^#^ Median (IQR).

* P-value was calculated for stable and exacerbations (E0) comparison using t-test unless stated below.

^‡^ Mann-Whitney-Wilcoxon Test.

^¶^ Data only collected for stable and six months visits.

FEV_1_, forced expiratory volume in one second; FVC, forced vital capacity.

**Table S2.** The OTUs detected in the negative reagent control with >10 sequencing reads.

| **OTU ID** | **# of Reads** | **Taxonomy** |
| --- | --- | --- |
| 663226 | 64 | k__Bacteria; p__Firmicutes; c__Clostridia; o__Clostridiales; f__Veillonellaceae; g__; s__ |
| 296082 | 24 | k__Bacteria; p__Bacteroidetes; c__Bacteroidia; o__Bacteroidales; f__Prevotellaceae; g__Prevotella; s__ |
| 856235 | 16 | k__Bacteria; p__Bacteroidetes; c__Bacteroidia; o__Bacteroidales; f__Prevotellaceae; g__Prevotella; s__ |
| 4318208 | 14 | k__Bacteria; p__Bacteroidetes; c__Bacteroidia; o__Bacteroidales; f__Prevotellaceae; g__Prevotella; s__copri |
| 4369988 | 12 | k__Bacteria; p__Firmicutes; c__Bacilli; o__Lactobacillales; f__Streptococcaceae; g__Streptococcus; s__ |
| 290370 | 11 | k__Bacteria; p__WPS-2; c__; o__; f__; g__; s__ |
| 516366 | 11 | k__Bacteria; p__Bacteroidetes; c__Bacteroidia; o__Bacteroidales; f__; g__; s__ |

**Table S3.** Occurrence and average relative abundance of contaminate genera detected in sequenced negative ‘blank’ controls by Salter et al.([2](#_ENREF_2)) in our microbiome data. The first column (occurrence rel abundance>0) was calculated as the fraction of samples in which each genus has abundance greater than 0. The second column (occurrence rel abundance>0.1) was calculated as the fraction of samples in which each genus has abundance greater than 0.1. And the third column is the average relative abundance of each genus across all samples.

| **Genus** | **Occurrence (rel abundance > 0)** | **Occurrence (rel abundance > 0.1)** | | **Average relative abundance** |
| --- | --- | --- | --- | --- |
| **Alphaproteobacteria** |  |  |  | |
| *Afipia* | 0 | 0 | 0 | |
| *Aquabacterium* | 0 | 0 | 0 | |
| *Asticcacaulis* | 0 | 0 | 0 | |
| *Aurantimonas* | 0 | 0 | 0 | |
| *Beijerinckia* | 0 | 0 | 0 | |
| *Bosea* | 0 | 0 | 0 | |
| *Bradyhizobium* | 0 | 0 | 0 | |
| *Brevundimonas* | 0.009709 | 0 | 5.43E-07 | |
| *Caulobacter* | 0 | 0 | 0 | |
| *Craurococcus* | 0 | 0 | 0 | |
| *Devosia* | 0.009709 | 0 | 1.09E-07 | |
| *Hoeflea* | 0 | 0 | 0 | |
| *Mesorhizobium* | 0 | 0 | 0 | |
| *Methylobacterium* | 0.145631 | 0 | 5.97E-06 | |
| *Novosphingobioum* | 0 | 0 | 0 | |
| *Ochrobactrum* | 0.009709 | 0 | 2.39E-06 | |
| *Paracoccus* | 0.038835 | 0 | 2.50E-06 | |
| *Pedomicrobiom* | 0 | 0 | 0 | |
| *Phyllobacterium* | 0 | 0 | 0 | |
| *Rhizobium* | 0.067961 | 0 | 1.84E-06 | |
| *Roseomonas* | 0 | 0 | 0 | |
| *Sphingobium* | 0.029126 | 0 | 1.09E-06 | |
| *Sphingomonas* | 0.291262 | 0 | 2.64E-05 | |
| *Sphingopyxis* | 0 | 0 | 0 | |
| **Betaproteobacteria** |  |  |  | |
| *Acidovorax* | 0.019417 | 0 | 2.17E-07 | |
| *Azoarcus* | 0 | 0 | 0 | |
| *Azospira* | 0 | 0 | 0 | |
| *Burkholderia* | 0 | 0 | 0 | |
| *Comamonas* | 0 | 0 | 0 | |
| *Cupriavidus* | 0.019417 | 0 | 1.19E-06 | |
| *Curvibacter* | 0 | 0 | 0 | |
| *Delftia* | 0.407767 | 0 | 5.58E-05 | |
| *Duganella* | 0 | 0 | 0 | |
| *Herbaspirillum* | 0 | 0 | 0 | |
| *Janthinobacterium* | 0.097087 | 0 | 9.88E-06 | |
| *Kingella* | 0.669903 | 0 | 0.000283 | |
| *Leptothrix* | 0 | 0 | 0 | |
| *Limnobacter* | 0 | 0 | 0 | |
| *Massilia* | 0 | 0 | 0 | |
| *Methylophilus* | 0 | 0 | 0 | |
| *Methyloversatilis* | 0.029126 | 0 | 4.34E-07 | |
| *Oxalobacter* | 0 | 0 | 0 | |
| *Pelomonas* | 0 | 0 | 0 | |
| *Polaromonas* | 0 | 0 | 0 | |
| *Ralstonia* | 0.019417 | 0 | 1.18E-05 | |
| *Schlegelella* | 0 | 0 | 0 | |
| *Sulfuritalea* | 0 | 0 | 0 | |
| *Undibacterium* | 0 | 0 | 0 | |
| *Variovorax* | 0 | 0 | 0 | |
| **Gammaproteobacteria** |  |  |  | |
| *Acinetobacter* | 0.184466 | 0 | 5.88E-05 | |
| *Enhydrobacter* | 0.029126 | 0 | 4.12E-06 | |
| *Enterobacter* | 0 | 0 | 0 | |
| *Escherichia* | 0 | 0 | 0 | |
| *Nevskia* | 0 | 0 | 0 | |
| *Pseudomonas* | 0.320388 | 0 | 7.04E-05 | |
| *Pseudoxanthomonas* | 0 | 0 | 0 | |
| *Psychobacter* | 0 | 0 | 0 | |
| *Stenotrophomonas* | 0.048544 | 0 | 2.39E-06 | |
| *Xanthomonas* | 0 | 0 | 0 | |
| **Actinobacteria**  *Aeromicrobium* | 0.009709 | 0 | 1.30E-06 | |
| *Arthrobacter* | 0.019417 | 0 | 6.51E-07 | |
| *Beutenbergia* | 0 | 0 | 0 | |
| *Brevibacterium* | 0.038835 | 0 | 6.29E-06 | |
| *Corynebacterium* | 0.815534 | 0 | 0.000407 | |
| *Curtobacterium* | 0.009709 | 0 | 3.26E-07 | |
| *Dietzia* | 0.019417 | 0 | 1.74E-06 | |
| *Geodermatophilus* | 0 | 0 | 0 | |
| *Janibacter* | 0 | 0 | 0 | |
| *Kocuria* | 0.135922 | 0 | 4.56E-06 | |
| *Microbacterium* | 0 | 0 | 0 | |
| *Micrococcus* | 0.097087 | 0 | 1.44E-05 | |
| *Microlunatus* | 0 | 0 | 0 | |
| *Patulibacter* | 0 | 0 | 0 | |
| *Propionibacterum* | 0 | 0 | 0 | |
| *Rhodococcus* | 0.009709 | 0 | 7.60E-07 | |
| *Tsukamurella* | 0 | 0 | 0 | |
| **Firmicutes** |  |  |  | |
| *Abiotrophia* | 0 | 0 | 0 | |
| *Bacillus* | 0.135922 | 0 | 2.06E-06 | |
| *Brevibacillus* | 0 | 0 | 0 | |
| *Brochothrix* | 0 | 0 | 0 | |
| *Facklamia* | 0 | 0 | 0 | |
| *Paenibacillus* | 0.009709 | 0 | 3.26E-07 | |
| *Streptococcus* | 1 | 0.281553 | 0.085535 | |
| **Bacteroidetes** |  |  |  | |
| *Chryseobacterium* | 0.067961 | 0 | 4.38E-05 | |
| *Dyadobacter* | 0.019417 | 0 | 2.17E-07 | |
| *Flavobacterium* | 0.038835 | 0 | 8.68E-07 | |
| *Hydrotalea* | 0 | 0 | 0 | |
| *Niatella* | 0 | 0 | 0 | |
| *Olivibacter* | 0 | 0 | 0 | |
| *Pedobacter* | 0.038835 | 0 | 1.63E-06 | |
| *Wautersiella* | 0.019417 | 0 | 4.12E-06 | |
| **Deinococcus-Thermus** |  |  |  | |
| *Deinococcus* | 0.029126 | 0 | 2.50E-06 | |

**Table S4.** Associations of the 10 most abundant microbiome genera, Shannon diversity, tPCs and pPCs with clinical metadata, including COPD disease status, patient subtypes, CAT score and lung function measurements. FDR *P*-values were indicated in the table. H: healthy, S: stable, E0: exacerbations, E2: two week post-exacerbations, E6: six week post-exacerbations, 6m: six months from first stable, HNS: healthy non-smokers, HS: healthy smokers, CS: COPD current smokers, ES: COPD ex-smokers, IE: infrequent exacerbators, FE: frequent exacerbators. *** FDR *P* < 0.001, ** FDR *P* < 0.01, * FDR *P* < 0.05.

| **Microbiome** | **Time points** | | | | | **GOLD Status** | | | **Smoking history** | | **ICS** | **Exac Freq** | **CAT** | **Lung function** | | |
| --- | --- | --- | --- | --- | --- | --- | --- | --- | --- | --- | --- | --- | --- | --- | --- | --- |
|  | H vs S | S vs E0 | E0 vs E2 | E2 vs E6 | E6 vs 6m | 1 vs 2 | 2 vs 3 | 3 vs 4 | HNS vs HS | CS vs ES | Yes vs No | IE vs FE | CAT | Post-FEV_1_ | Post-FVC | FEV_1_/FVC Ratio |
| *Moraxella* | 0.02* | 0.02* | 0.01** | 0.47 | 0.02* | 0.99 | 0.00*** | 0.06 | 0.25 | 0.99 | 0.02* | 0.98 | 0.89 | 0.52 | 0.65 | 0.86 |
| *Neisseria* | 0.51 | 0.02* | 0.99 | 0.01* | 0.02* | 0.99 | 0.99 | 0.99 | 0.31 | 0.28 | 0.99 | 0.58 | 0.09 | 0.06 | 0.72 | 0.21 |
| *Haemophilus* | 0.99 | 0.99 | 0.99 | 0.99 | 0.99 | 0.99 | 0.82 | 0.95 | 0.04* | 0.03* | 0.21 | 0.71 | 0.30 | 0.31 | 0.76 | 0.86 |
| *Streptococcus* | 0.00** | 0.35 | 0.99 | 0.96 | 0.69 | 0.99 | 0.65 | 0.87 | 0.99 | 0.09 | 0.99 | 0.41 | 0.79 | 0.91 | 0.65 | 0.86 |
| *Prevotella* | 0.59 | 0.99 | 0.99 | 0.97 | 0.92 | 0.99 | 0.99 | 0.99 | 0.82 | 0.96 | 0.32 | 0.83 | 0.30 | 0.20 | 0.65 | 0.86 |
| *Veillonella* | 0.99 | 0.88 | 0.99 | 0.99 | 0.99 | 0.99 | 0.99 | 0.95 | 0.99 | 0.99 | 0.99 | 0.98 | 0.44 | 0.91 | 0.82 | 0.95 |
| *Megasphaera* | 0.81 | 0.99 | 0.99 | 0.96 | 0.65 | 0.99 | 0.58 | 0.99 | 0.86 | 0.28 | 0.82 | 0.99 | 0.79 | 0.89 | 0.76 | 0.72 |
| *Selenomonas* | 0.78 | 0.51 | 0.99 | 0.85 | 0.65 | 0.99 | 0.99 | 0.55 | 0.99 | 0.99 | 0.13 | 0.30 | 0.89 | 0.11 | 0.33 | 0.86 |
| S24-7 | 0.35 | 0.31 | 0.26 | 0.10 | 0.40 | 0.99 | 0.99 | 0.50 | 0.60 | 0.99 | 0.22 | 0.85 | 0.79 | 0.30 | 0.57 | 0.86 |
| Shannon | 0.03* | 0.12 | 0.36 | 0.17 | 0.98 | 0.69 | 0.89 | 0.62 | 0.67 | 0.19 | 0.09 | 0.43 | 0.07 | 0.21 | 0.14 | 0.11 |
| tPC1 | NA | 0.01* | 0.87 | 0.90 | 0.90 | 0.73 | 0.80 | 0.99 | NA | 0.59 | 0.53 | 0.14 | 0.83 | 0.96 | 0.82 | 0.97 |
| tPC2 | NA | 0.89 | 0.87 | 0.90 | 0.81 | 0.51 | 0.80 | 0.99 | NA | 0.80 | 0.53 | 0.99 | 0.79 | 0.96 | 0.82 | 0.97 |
| tPC3 | NA | 0.82 | 0.90 | 0.90 | 0.81 | 0.51 | 0.99 | 0.99 | NA | 0.80 | 0.79 | 0.99 | 0.56 | 0.96 | 0.82 | 0.97 |
| tPC4 | NA | 0.04* | 0.87 | 0.99 | 0.90 | 0.51 | 0.99 | 0.99 | NA | 0.80 | 0.79 | 0.99 | 0.92 | 0.96 | 0.94 | 0.97 |
| tPC5 | NA | 0.58 | 0.99 | 0.99 | 0.99 | 0.96 | 0.80 | 0.99 | NA | 0.70 | 0.53 | 0.99 | 0.83 | 0.89 | 0.82 | 0.97 |
| tPC6 | NA | 0.31 | 0.99 | 0.90 | 0.26 | 0.99 | 0.80 | 0.99 | NA | 0.80 | 0.99 | 0.99 | 0.83 | 0.96 | 0.82 | 0.97 |
| tPC7 | NA | 0.89 | 0.87 | 0.90 | 0.81 | 0.51 | 0.80 | 0.99 | NA | 0.80 | 0.53 | 0.99 | 0.83 | 0.96 | 0.82 | 0.97 |
| tPC8 | NA | 0.17 | 0.57 | 0.90 | 0.96 | 0.73 | 0.80 | 0.99 | NA | 0.80 | 0.55 | 0.21 | 0.79 | 0.96 | 0.82 | 0.97 |
| tPC9 | NA | 0.54 | 0.99 | 0.90 | 0.26 | 0.51 | 0.99 | 0.99 | NA | 0.59 | 0.53 | 0.14 | 0.56 | 0.89 | 0.82 | 0.97 |
| pPC1 | NA | 0.21 | 0.27 | 0.80 | 0.38 | 0.65 | 0.67 | 0.99 | NA | 0.01** | 0.93 | 0.67 | 0.41 | 0.50 | 0.95 | 0.79 |
| pPC2 | NA | 0.21 | 0.27 | 0.40 | 0.51 | 0.65 | 0.67 | 0.99 | NA | 0.62 | 0.46 | 0.99 | 0.65 | 0.88 | 0.35 | 0.56 |
| pPC3 | NA | 0.20 | 0.27 | 0.40 | 0.98 | 0.65 | 0.67 | 0.99 | NA | 0.62 | 0.46 | 0.67 | 0.41 | 0.71 | 0.66 | 0.53 |
| pPC4 | NA | 0.91 | 0.84 | 0.80 | 0.76 | 0.65 | 0.67 | 0.99 | NA | 0.38 | 0.46 | 0.99 | 0.65 | 0.50 | 0.95 | 0.45 |
| pPC5 | NA | 0.91 | 0.84 | 0.40 | 0.99 | 0.65 | 0.67 | 0.99 | NA | 0.70 | 0.46 | 0.99 | 0.90 | 0.50 | 0.03* | 0.99 |
| pPC6 | NA | 0.99 | 0.84 | 0.40 | 0.76 | 0.86 | 0.67 | 0.99 | NA | 0.11 | 0.46 | 0.67 | 0.65 | 0.71 | 0.49 | 0.20 |
| pPC7 | NA | 0.21 | 0.84 | 0.80 | 0.38 | 0.65 | 0.70 | 0.99 | NA | 0.87 | 0.46 | 0.99 | 0.90 | 0.50 | 0.56 | 0.24 |
| pPC8 | NA | 0.83 | 0.84 | 0.64 | 0.76 | 0.65 | 0.67 | 0.99 | NA | 0.11 | 0.46 | 0.67 | 0.90 | 0.33 | 0.95 | 0.24 |

**Table S5.** Spearman correlations between sputum cell counts and percentage and the 10 most abundant microbiome genera, Shannon diversity, tPCs and pPCs across all samples. The FDR *P*-values are indicated in the table. Significant positive correlations are highlighted in red. Significant negative correlations are highlighted in blue.

| **Microbiome/ tPCs/pPCs** | **Eosin-ophil** | **Epithe-lial** | **Lymphocyte** | **Macro-phage** | **Neutr-ophil** | **Eosin%** | **Epith %** | **Lymp%** | **Macro %** | **Neutr%** |
| --- | --- | --- | --- | --- | --- | --- | --- | --- | --- | --- |
| *Prevotella* | 0.994 | 0.380 | 0.023 | 0.975 | 0.227 | 0.767 | 0.746 | 0.031 | 0.064 | 0.034 |
| S24-7 | 0.871 | 0.380 | 0.783 | 0.975 | 0.942 | 0.767 | 0.490 | 0.750 | 0.697 | 0.577 |
| *Streptococcus* | 0.871 | 0.249 | 0.783 | 0.346 | 0.008 | 0.152 | 0.061 | 0.385 | 0.013 | 8E-3 |
| *Megasphaera* | 0.673 | 0.740 | 0.778 | 0.120 | 0.004 | 0.767 | 2E-3 | 0.365 | 0.111 | 0.020 |
| *Selenomonas* | 0.871 | 0.869 | 0.783 | 0.975 | 0.394 | 0.767 | 0.490 | 0.990 | 0.524 | 0.267 |
| *Veillonella* | 0.871 | 0.740 | 0.452 | 0.438 | 0.012 | 0.427 | 4E-3 | 0.312 | 0.064 | 6E-3 |
| *Neisseria* | 0.871 | 0.740 | 0.783 | 0.646 | 0.049 | 0.767 | 0.073 | 0.674 | 0.028 | 7E-3 |
| *Haemophilus* | 0.871 | 0.737 | 0.672 | 0.646 | 0.029 | 0.767 | 0.011 | 0.312 | 0.013 | 6E-3 |
| *Moraxella* | 0.871 | 0.502 | 0.672 | 0.646 | 0.142 | 0.767 | 0.065 | 0.385 | 0.336 | 0.071 |
| Shannon | 0.968 | 0.249 | 0.783 | 0.975 | 0.156 | 0.685 | 0.491 | 0.397 | 6E-3 | 0.023 |
| tPC1 | 0.231 | 0.508 | 0.019 | 0.895 | 1E-4 | 1E-3 | 0.162 | 3E-4 | 4E-9 | 7E-10 |
| tPC2 | 0.867 | 0.985 | 0.980 | 0.716 | 0.411 | 0.973 | 0.247 | 0.696 | 0.336 | 0.036 |
| tPC3 | 0.867 | 0.985 | 0.980 | 0.329 | 0.948 | 0.363 | 0.958 | 0.841 | 0.798 | 0.801 |
| tPC4 | 0.867 | 0.515 | 0.980 | 0.043 | 0.006 | 0.973 | 0.138 | 0.696 | 0.613 | 0.196 |
| tPC5 | 0.867 | 0.473 | 0.980 | 0.716 | 0.510 | 0.973 | 0.162 | 0.696 | 0.914 | 0.486 |
| tPC6 | 0.994 | 0.985 | 0.980 | 0.716 | 0.948 | 0.973 | 0.958 | 0.696 | 0.700 | 0.796 |
| tPC7 | 0.867 | 0.985 | 0.980 | 0.895 | 0.510 | 0.973 | 0.575 | 0.841 | 0.700 | 0.433 |
| tPC8 | 0.867 | 0.508 | 0.980 | 0.297 | 0.819 | 0.973 | 0.958 | 0.951 | 0.798 | 0.850 |
| tPC9 | 0.867 | 0.508 | 0.980 | 0.716 | 0.510 | 0.973 | 0.142 | 0.696 | 0.298 | 0.196 |
| pPC1 | 0.867 | 0.593 | 0.366 | 0.471 | 2E-3 | 0.121 | 0.194 | 0.221 | 0.026 | 0.852 |
| pPC2 | 0.867 | 0.593 | 0.366 | 0.590 | 6E-4 | 0.121 | 0.042 | 0.160 | 1E-3 | 4E-4 |
| pPC3 | 0.883 | 0.593 | 0.366 | 0.284 | 0.151 | 0.170 | 0.888 | 0.405 | 0.860 | 5E-3 |
| pPC4 | 0.687 | 0.613 | 0.366 | 0.303 | 0.375 | 0.170 | 0.888 | 0.751 | 0.860 | 0.747 |
| pPC5 | 0.687 | 0.593 | 0.785 | 0.708 | 0.552 | 0.416 | 0.888 | 0.387 | 0.860 | 0.724 |
| pPC6 | 0.687 | 0.637 | 0.366 | 0.471 | 0.552 | 0.416 | 0.888 | 0.387 | 0.900 | 0.885 |
| pPC7 | 0.687 | 0.593 | 0.366 | 0.303 | 0.383 | 0.170 | 0.888 | 0.387 | 0.860 | 0.753 |
| pPC8 | 0.687 | 0.613 | 0.366 | 0.208 | 0.151 | 0.121 | 0.321 | 0.387 | 0.860 | 0.421 |

**Table S6.** Spearman correlations between qPCR quantities of total bacterial load and five bacterial species and sputum cell counts or percentage, tPCs and pPCs across all samples. The FDR *P*-values are indicated in the table. Significant positive correlations are highlighted in red. Significant negative correlations are highlighted in blue. Pan: total bacterial load, HI: *Haemophilus influenzae*, MC: *Moraxella catarrhalis*, SP: *Streptococcus pneumoniae*, PM: *Prevotella melaninogenica*, VD: *Veillonella dispar*.

| **Cell counts or percentage/tPCs/pPCs** | **Log Pan** | **Log HI** | **Log MC** | **Log SP** | **Log PM** | **Log VD** |
| --- | --- | --- | --- | --- | --- | --- |
| Eosinophil | 0.473 | 0.950 | 0.788 | 0.298 | 0.618 | 0.745 |
| Epithelial | 0.135 | 0.603 | 0.832 | 0.013 | 0.387 | 0.393 |
| Lymphocyte | 0.613 | 0.107 | 0.096 | 0.249 | 0.125 | 0.430 |
| Macrophage | 0.361 | 0.663 | 0.098 | 0.310 | 0.171 | 0.438 |
| Neutrophil | 0.446 | 0.037 | 0.003 | 0.098 | 0.047 | 0.338 |
| Eosino % | 0.444 | 0.360 | 0.110 | 0.889 | 0.846 | 0.827 |
| Epith % | 0.561 | 0.112 | 0.011 | 0.951 | 0.163 | 0.051 |
| Lympho % | 0.702 | 0.061 | 0.046 | 0.184 | 0.145 | 0.352 |
| Macro % | 0.340 | 0.004 | 0.006 | 0.403 | 0.531 | 0.894 |
| Neutro % | 0.434 | 0.003 | 3E-4 | 0.578 | 0.267 | 0.481 |
| tPC1 | 0.904 | 0.118 | 0.069 | 0.201 | 0.651 | 0.476 |
| tPC2 | 0.035 | 0.017 | 0.859 | 0.246 | 0.052 | 0.087 |
| tPC3 | 0.214 | 0.194 | 0.342 | 0.309 | 0.777 | 0.544 |
| tPC4 | 0.184 | 0.621 | 0.046 | 0.087 | 0.987 | 0.130 |
| tPC5 | 0.630 | 0.441 | 0.544 | 0.147 | 0.401 | 0.418 |
| tPC6 | 0.837 | 0.171 | 0.680 | 0.121 | 0.043 | 0.962 |
| tPC7 | 0.772 | 0.513 | 0.160 | 0.753 | 0.631 | 0.790 |
| tPC8 | 0.942 | 0.729 | 0.900 | 0.409 | 0.300 | 0.251 |
| tPC9 | 0.782 | 0.001 | 0.142 | 0.043 | 0.035 | 0.904 |
| pPC1 | 0.705 | 0.204 | 0.092 | 0.196 | 0.655 | 0.153 |
| pPC2 | 0.277 | 0.631 | 0.119 | 0.007 | 0.013 | 0.881 |
| pPC3 | 0.599 | 0.003 | 0.090 | 0.136 | 0.324 | 0.865 |
| pPC4 | 0.340 | 0.825 | 0.689 | 0.455 | 0.500 | 0.872 |
| pPC5 | 0.298 | 0.766 | 0.789 | 0.184 | 0.511 | 0.531 |
| pPC6 | 0.251 | 0.597 | 0.222 | 0.785 | 0.384 | 0.057 |
| pPC7 | 0.347 | 0.553 | 0.327 | 0.829 | 0.131 | 0.728 |
| pPC8 | 0.997 | 0.404 | 0.585 | 0.435 | 0.007 | 0.240 |

**References**

1. Robinson MD, McCarthy DJ, Smyth GK. edgeR: a Bioconductor package for differential expression analysis of digital gene expression data. Bioinformatics. 2010;26(1):139-40.

2. Salter SJ, Cox MJ, Turek EM, Calus ST, Cookson WO, Moffatt MF, et al. Reagent and laboratory contamination can critically impact sequence-based microbiome analyses. BMC biology. 2014;12:87.
